# Sequence Variation within the KIV-2 Copy Number Polymorphism of the Human *LPA* Gene in African, Asian, and European Populations

**DOI:** 10.1371/journal.pone.0121582

**Published:** 2015-03-30

**Authors:** Asma Noureen, Friedrich Fresser, Gerd Utermann, Konrad Schmidt

**Affiliations:** 1 Division of Genetic Epidemiology, Department of Medical Genetics, Molecular and Clinical Pharmacology, Innsbruck Medical University, Innsbruck, Austria; 2 Division of Human Genetics, Department of Medical Genetics, Molecular and Clinical Pharmacology, Innsbruck Medical University, Innsbruck, Austria; 3 Division of Translational Cell Genetics, Department of Medical Genetics, Molecular and Clinical Pharmacology, Innsbruck Medical University, Innsbruck, Austria; 4 Centre de Recherches Médicales de Lambaréné, Albert Schweitzer Hospital, Lambaréné, Gabon; 5 Department for Tropical Medicine, Eberhard-Karls-University Tübingen, Tübingen, Germany; University of Oxford, UNITED KINGDOM

## Abstract

Amazingly little sequence variation is reported for the kringle IV 2 copy number variation (KIV 2 CNV) in the human *LPA* gene. Apart from whole genome sequencing projects, this region has only been analyzed in some detail in samples of European populations. We have performed a systematic resequencing study of the exonic and flanking intron regions within the KIV 2 CNV in 90 alleles from Asian, European, and four different African populations. Alleles have been separated according to their CNV length by pulsed field gel electrophoresis prior to unbiased specific PCR amplification of the target regions. These amplicons covered all KIV 2 copies of an individual allele simultaneously. In addition, cloned amplicons from genomic DNA of an African individual were sequenced. Our data suggest that sequence variation in this genomic region may be higher than previously appreciated. Detection probability of variants appeared to depend on the KIV 2 copy number of the analyzed DNA and on the proportion of copies carrying the variant. Asians had a high frequency of so-called KIV 2 type B and type C (together 70% of alleles), which differ by three or two synonymous substitutions respectively from the reference type A. This is most likely explained by the strong bottleneck suggested to have occurred when modern humans migrated to East Asia. A higher frequency of variable sites was detected in the Africans. In particular, two previously unreported splice site variants were found. One was associated with non-detectable Lp(a). The other was observed at high population frequencies (10% to 40%). Like the KIV 2 type B and C variants, this latter variant was also found in a high proportion of KIV 2 repeats in the affected alleles and in alleles differing in copy numbers. Our findings may have implications for the interpretation of SNP analyses in other repetitive loci of the human genome.

## Introduction

Lipoprotein(a) (Lp(a)) is a complex particle in human plasma. It is assembled from a low density lipoprotein (LDL) and a glycoprotein, apolipoprotein (apo(a)), which is encoded by the *LPA* gene (MIM +152200). Lp(a) levels in plasma range from <0.1 mg/dl to >100 mg/dl, but are highly heritable. The mean concentrations vary considerably between ethnic groups, with Africans exhibiting on average two- to threefold higher Lp(a) levels compared to Europeans. In all populations, *LPA* is the major gene controlling this quantitative and co-dominantly expressed trait. The physiological function of Lp(a)/apo(a) is still unknown, whereas its association with coronary heart disease is well established (for review see [1]).

**Fig 1:**
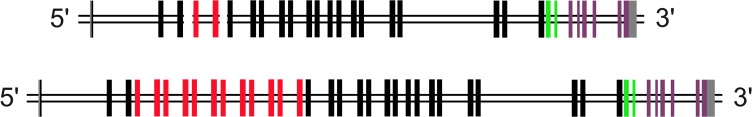
*LPA* gene structure. The *LPA* gene contains 27 non-repetitive exons including the 5’UTR (grey), one copy each of kringle (K) domains KIV-1, KIV-3 to KIV-10 (black), KV (green), all comprising two exons, followed by six exons encoding the protease-like domain (purple) and the 3’UTR (grey). The KIV-2 domain (red) varies in number from 1 to >40 copies, thereby forming the KIV-2 CNV. Each KIV-2 repeat is approximately 5.5 kb long, composed of the first exon (160 bp), a long intron (4 kb), the second exon (182 bp), and a short intron (1.2 kb). Depicted is the general exon/intron structure of the *LPA* gene, with one KIV-2 repeat, and an example containing six KIV-2 repeats and, hence, 15 KIV repeats (as is the case for *LPA* in the human reference sequence).

*LPA* shares a high degree of homology with plasminogen (*PLG*) from which it evolved by deletion, gene duplication and gene conversion events during primate evolution [2]. A distinctive feature of *LPA* is the presence of so called kringle domains. While the kringles I, II, and III of *PLG* are missing in primate *LPA*, the kringle IV domain has been multiplied and diversified. Thus human *LPA* is composed of 10 different types of kringle IV repeats (KIV-1 to KIV-10), one kringle V (KV), and a protease-like domain. The KIV-1 and KIV-3 to KIV-10 domains are each present in a single copy, whereas KIV-2 shows an extensive copy number variation (CNV), ranging from 1 to >40 copies (Fig. 1). Each KIV-2 copy is 5.5 kb in size and consists of two exons of 160 bp respectively 182 bp, which are linked by a large intron (4 kb), and a short intron (1.2 kb) that joins it to the next copy.

**Fig 2:**
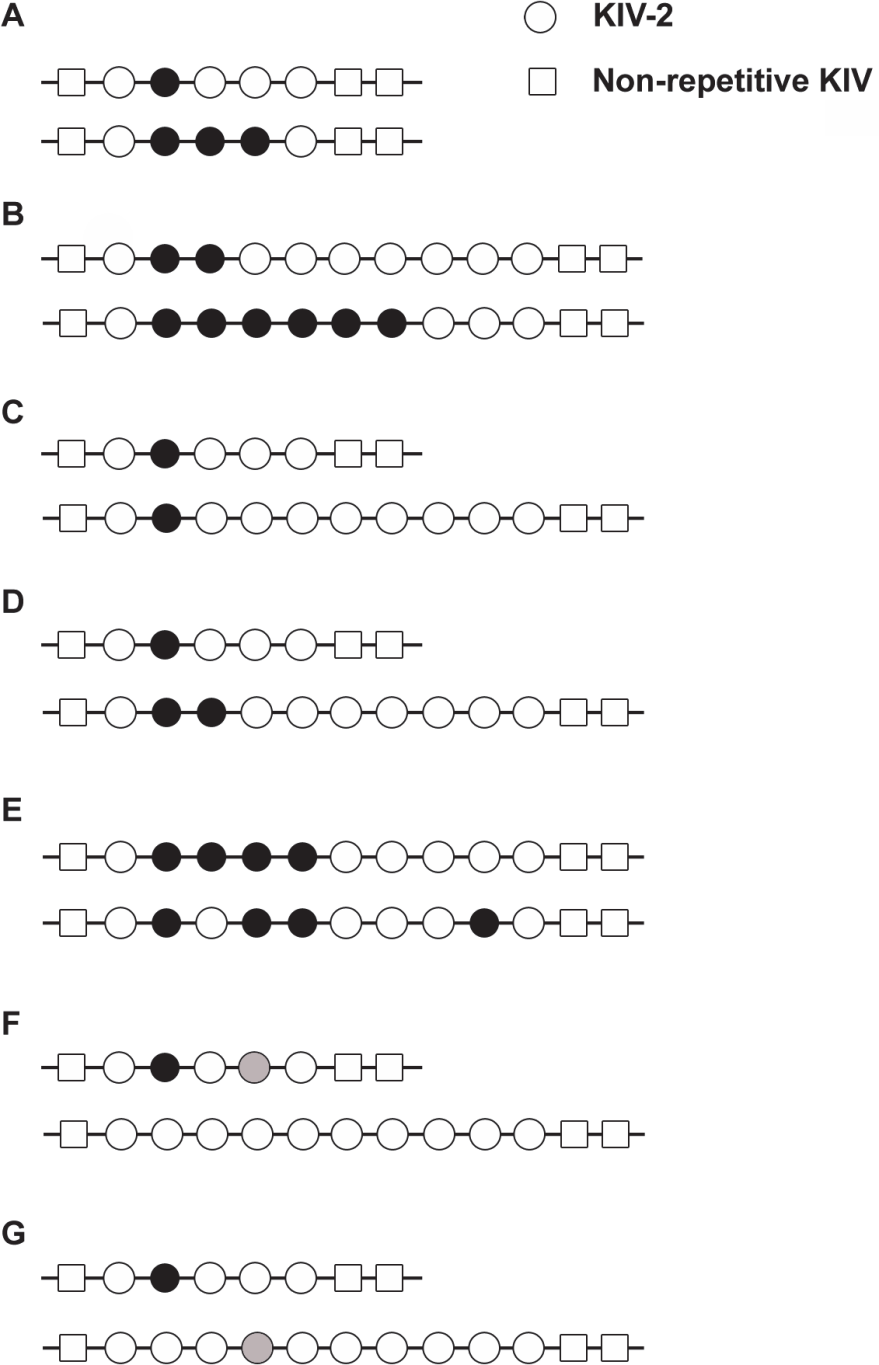
Examples for the possible distribution of sequence variations in KIV-2 CNV alleles. Sequence variants affecting KIV-2 copies (shown as filled circles) can be present in single or several KIV-2 copies within alleles of different sizes. (A) Low and high intra-allelic frequencies of a variant on short (e.g. 5 KIV-2) alleles. (B) Low and high intra-allelic frequencies of a variant for longer (limited to 10 KIV-2 for graphic display) alleles. (C) The same number of KIV-2 copies harbors the variant on a short and a longer allele, i.e. the intra-allelic frequency of the variant is higher on the short allele. Thus detection of the variant would be more likely if present on the short allele. (D) Both alleles have the same intra-allelic frequency (20%), though the number of copies carrying the variant is different. Hence the probability of detection would be the same. (E) The order of variant carrying KIV-2 copies within the allele might vary. These different scenarios cannot be distinguished by present methods. (F) Different variants can be allocated in *cis*, shown for a genotype with one short and one longer allele or (G) in *trans*. While scenarios F and G cannot be distinguished in analyses based on diploid samples, this is possible in our analysis based on separated alleles.

The extensive size variation of KIV-2 alleles results in a high degree of heterozygosity within populations. The KIV-2 CNV is a major causal determinant of Lp(a) levels [3,4]. A general inverse correlation exists between the KIV-2 CNV size and the associated Lp(a) plasma concentrations. However, there are notable differences between populations in the strength of this inverse correlation [5]. Also, a large variation of Lp(a) concentrations is observed for alleles with identical KIV-2 CNV size [6–8]. It has been demonstrated that sequence variation in *LPA* other than the KIV-2 CNV is also responsible for variability in Lp(a) levels. To date, several single nucleotide polymorphisms (SNPs) in *LPA* have been shown to be associated with Lp(a) concentrations, and for some a causal mechanism has been established, e.g. by effects on splicing [9], nonsense mutation [10], or by introduction of an additional ATG start codon [11]. Most of this variation is in the so-called unique (i.e. non-repetitive) kringles, the protease domain, and in regulatory sequences. However, for the region of the KIV-2 CNV, comparatively little is known about other sequence variation than the size polymorphism itself. This poses a gap in our understanding of *LPA* respectively the apo(a)/Lp(a) trait, as the multitude of KIV-2 copies might render any phenotypically important amino-acid variation therein difficult to detect. According to the human reference sequence, the paralogous KIV-2 copies are 98% to 100% homologous in the first exons (depending on their “type”, A or B, see below) and 100% in the second exons, respectively. In principle, paralogous variation within KIV-2 units can include high frequency variants and a multitude of different rare variants. Also variants may be present on a single, several or all KIV-2 repeats of an individual allele (intra-allelic variation), all of either high or low population frequencies (Fig. 2). Variation found on several copies of individual alleles can be considered not to derive by *de novo* point mutations as assumed for conventional SNPs, but rather from duplication events or from gene conversion of highly homologous sequences, in this particular case within the KIV-2 CNV, and might be therefore classified as paralogous sequence variants (PSVs). Further, any variation may be present on short or long KIV-2 alleles. Some of this variation may be population specific, and even contribute to explain differences in Lp(a) levels between populations or result in functional properties restricted to some populations.

Previous studies suggest that in Europeans, KIV-2 repeats show little population or intra-allelic sequence variation [10,12,13]. Three types of KIV-2 repeats designated A, B, and C, which differ in three or two exonic positions respectively on the same allele have been described [2,10]. Type B is defined by synonymous substitutions at the bases 14 (A>G), 41 (T>C) and 86 (A>T) of the KIV-2 exon 1 domains, while type C only harbors the substitutions at bases 14 and 41. In the human reference sequence, all but one of the six KIV-2 exon 1 domains are of type A; only the third one is of type B. Only a few non-Europeans have been analyzed systematically for sequence variation in the KIV-2 repeats of *LPA*. The aim of our study therefore was to resequence this repeat domain to find potentially functionally significant sequence variation in populations from Africa and Asia, and to compare them with Europeans analyzed by the same standards.

Our approach was mainly based upon batchwise sequencing of PCR amplified KIV-2 fragments from individual *LPA* alleles, which had been separated by pulsed field gel electrophoresis (PFGE) according to the size of their KIV-2 CNV. This also allowed to directly access the association of KIV-2 allele sizes with sequence variation within this domain (Fig. 2, panel F and G). Cloned KIV-2 fragments from two alleles of a South African Bantu individual were analyzed for low intra-allelic frequency variation, and to assess the sensitivity of the batchwise technique.

## Materials and Methods

### Ethics statement

All samples were completely anonymized prior to inclusion in the analyses, and the analyses were performed in compliance with the Austrian Gene Technology Act, the federal law regulating genetic research in Austria. Informed consent was obtained from all participants during the sampling process, in writing where possible. In populations with a high percentage of illiterate participants, the study objectives and procedures were explained in their respective languages in understandable terms and the participants then gave verbal consent. Consent was documented by the investigator. The consent procedures and the study design were approved by the ethics committee of Innsbruck Medical University, Austria (reference UN4485, 305/4.1).

### Population samples

Samples were selected randomly from a larger sample base previously collected in six populations from three geographical regions, namely Africa (Khoi San, South African Bantu, Gabonese Bantu, Egyptians), Europe (Austrians), and Asia (Hong Kong Chinese). The original blood samples of unrelated, apparently healthy individuals were drawn in Ismailia (Egypt, n = 127), Lambarene (Gabon, n = 116) and Pretoria (Republic of South Africa, RSA, n = 318). The Khoi San samples were also collected in Schmidtsdrift (RSA, n = 332). These people of Angolan descent had been relocated in 1990 in the aftermath of the South African Border War. The European participants were recruited in Tyrol (Austria), and the Asians in Hong Kong (China, n = 200). Information on the KIV-2 CNV size and Lp(a) plasma concentrations, measured by ELISA as described in [14], was available for all of these samples from prior analyses.

From this set of population samples, in total 45 samples were screened of sequence variation within the KIV-2 CNV: 10 Gabonese Bantu (IDs G1 to G10), Austrians (A1 to A10), and Hong Kong Chinese (H1 to H10) each, and 5 Khoi San (K1 to K5), South African Bantu (S1 to S5), and Egyptians (E1 to E5) each. To select these samples, the “random selection of samples” function in SPSS 20 was applied for each population, after limiting the possible choice to samples heterozygous at the KIV-2 CNV to allow for later allele separation (see below). Any subsample was then subjected to a non-parametric test (Mann-Whitney-U test and two sample Kolmogorov-Smirnov test) for no significant deviation in either the Lp(a) plasma concentration or KIV-2 CNV allele size frequency distributions to the respective total, unlimited population samples.

While a statistical analysis for the association of any sequence variants in the KIV-2 CNV with Lp(a) plasma concentrations is not possible with the comparatively small number of samples screened from each population, information on allele associated Lp(a) concentrations [15] was available from previously conducted Western Blots for some samples [16,17]. This allowed directly assessing the phenotypic impact of some specific variants. However, to avoid selection bias, unlike in some previous study [10], none of the samples included in our resequencing project was selected due to a specific Lp(a)/apo(a) phenotype.

### DNA extraction

DNA was extracted shortly after the sampling in a way to ensure that the samples are suitable for separation of the two *LPA* alleles by pulsed field gel electrophoresis (PFGE). At the sampling site, a total of 9 ml blood was drawn into EDTA-containing tubes, and plasma (1 ml) was separated by low speed centrifugation. These plasma samples and blood concentrates were frozen immediately and sent to Innsbruck on dry ice for further processing. Genomic DNA was extracted from white blood cells and embedded in low-melting point agarose as so-called “plugs” [18]. Since then, these DNA containing agarose plugs had been stored in 0.5 M EDTA solution at 4°C.

### Determination of the number of KIV-2 repeats and allele separation by PFGE after KpnI digestion

For all samples, KIV-2 copy numbers in the *LPA* gene had been determined prior to our sample selection for this study. In short, DNA-containing plugs were digested with the restriction enzyme KpnI and subjected to PFGE, followed by Southern blotting with a KIV-2-specific probe [18]. Because this previous KIV-2 CNV typing had been performed over a period of several years, we have now retyped all of the 45 samples selected for our study by PFGE (for exact protocols, see S1 Protocol), thus correcting for any previous inter-population evaluation bias. The number of KIV-2 repeats of individual alleles was determined by comparison to previously defined standards, for which the KIV-2 copy number was confirmed by fibre-FISH [19], and to a molecular size ladder (Lambda Ladder PFG Marker, New England Biolabs).

Each sample was run once for size determination and once for cutting the alleles. For cutting the alleles, two half plugs were digested according to the protocol given in Supplementary Online Information “Materials and Methods”. These were then applied on the same gel, which was split in two after PFGE (S1 Fig.). DNA from the cut alleles was extracted for downstream applications by centrifugation in Ultrafree-DA Centrifugal Unit tubes (Merck Millipore).

### Batchwise sequence analysis

According to the sequence data available so far from the Human Genomes Project and previous studies, the high inter kringle homology within the KIV-2 CNV should allow the amplification of KIV-2 specific sequences from all the KIV-2 copies of an allele in one PCR reaction, i.e. in a batch-wise manner. Care was taken to position the PCR primers (S1 Table) so that all other non-repetitive kringles, especially the highly similar KIV-1 exon 2 and KIV-3 exon 1, were not amplified. At the same time, all the KIV-2 repeats should be amplified unbiased, i.e. with no distinction between previously reported variants as KIV-2 exon 1 type A, B, or C. The high inter-domain homology within *LPA* together with the intra-domain differences inside the KIV-2 limited the positions suitable for PCR primer location.

**Fig 3:**
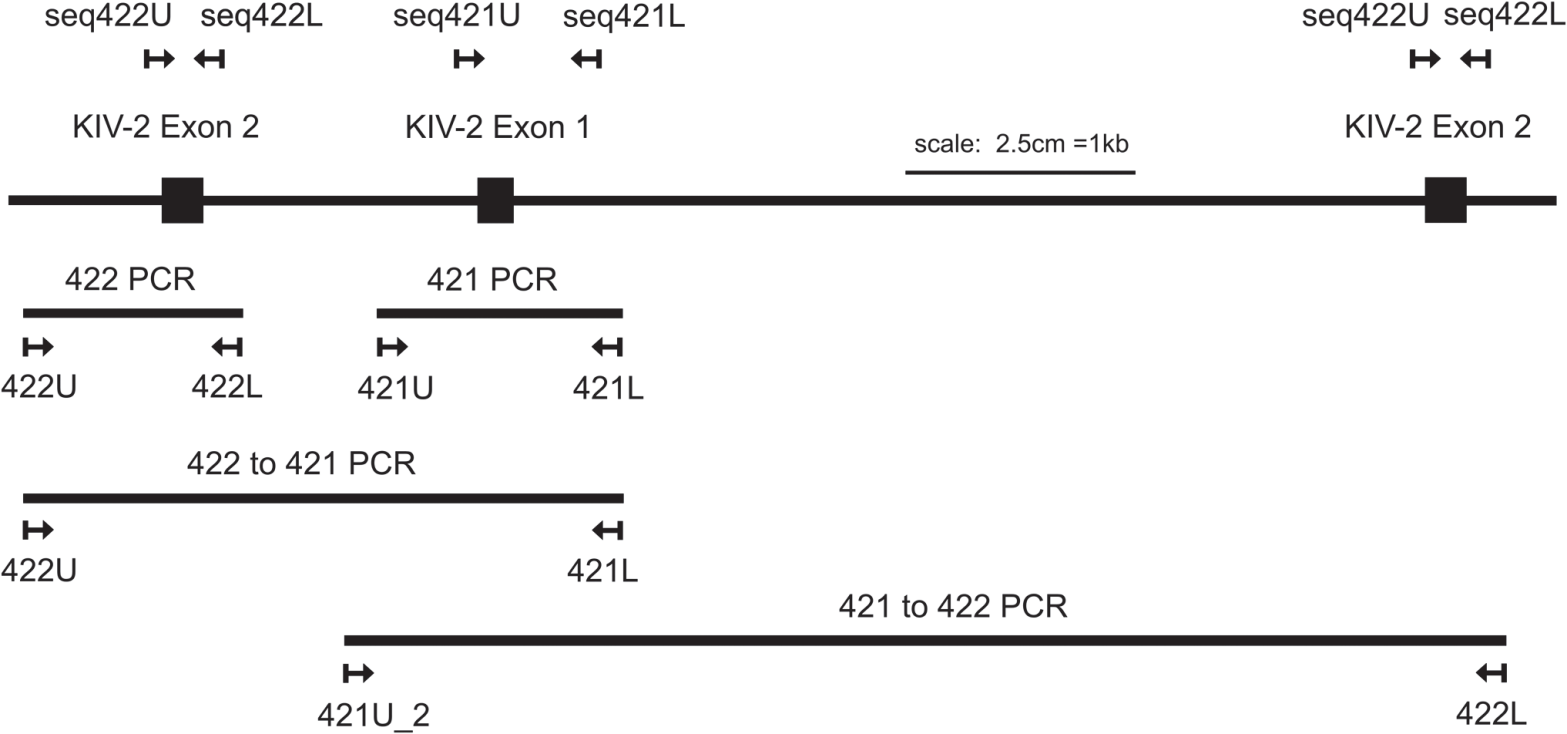
Positions of the PCR primers and regions amplified for cloning and batchwise screening. Below a stretch of KIV-2 domains showing the positions of the exons and the short and long introns in the KIV-2 CNV, the regions amplified by the different PCRs are depicted by horizontal lines with the names of the PCRs written above and the primers below. PCRs “421” and “422” are spanning the first and second exon of the KIV-2 respectively, and also contain the flanking intron sequences. These two PCRs were used in the batchwise screening and cloning. PCR “412to422” amplifies both exons and the long intron, while PCR “422to421” includes both exons and the short intron. The locations of the different primers used for sequencing are shown above the depicted stretch of KIV-2 CNV. The figure is drawn to scale (2.5 cm = 1 kb).

PCRs amplifying the regions 421 (1069 bp) and 422 (982 bp) (Fig. 3) were conducted according to the specific protocols given in S2 Table for all 90 separated alleles and one genomic sample. The same PCRs, with modified primers, were used for cloning (S1 Table). Amplification was controlled on 1% agarose in 1X TBE gel. Afterwards the fragments were purified using ExoSAP-IT (Affymetrix) according to the manufacturer's protocol before conducting sequence analysis.

Sequence analysis was restricted to the exon and immediate intron sequence for this project which was aimed at discovering variants with possible functional effects on the apo(a) protein, i.e. non-synonymous or splices site variation. Specifically, the sequence covered for all samples in unambiguous quality was reaching from 15 bp upstream to 100 bp downstream of KIV-2 exon 1, and from 30 bp upstream to 40 bp downstream of KIV-2 exon 2. Cycle sequencing and sequencing was conducted as outlined in S1 Protocol.

We assured specificity of our screening results further by analyses of the KIV-2 CNV flanking regions of KIV-1 exon 2 and KIV-3 exon 1 through specific PCRs for these regions (S3 Table).

### Cloning

Genomic DNA from one South African Bantu (KIV-2 CNV allele sizes of 18 and 32 repeats) was selected for KIV-2 exon screening by cloning. The primers for the PCR fragments 421 and 422 were modified to include the restriction sites at the 5’ ends. A SalI restriction site (GTCGAC) was introduced at the 5’ end of the upper primers and an EcoRV site (GATATC) was introduced at the 5’ end of the lower primers. An additional sequence of 6 bp (GGCGGC) was also added at the 5’ end to provide sufficient space for the restriction enzyme to efficiently bind and cut. PCRs were performed for a total volume of 100 ul using proofreading DNA polymerase Pfu (Fermentas) according to the conditions given in S2 Table and cloning was conducted as specified in the protocol given in S1 Protocol.

### Estimating the detection sensitivity of the batchwise screening

To determine the sensitivity of the batchwise technique and SNP detection threshold, a dilution experiment was performed. Two clones of the fragment 422 which were identified as carrying one SNP each, but at two different positions, were chosen for this purpose. The DNA concentration of the clones was measured by NanoDrop and adjusted to the same values. Afterwards both clones were mixed in different proportions, ranging from 0% to 100%, and sequenced with forward and reverse primers (S2 Fig.). For all samples, estimates for intra-allelic frequencies of variants are given as read by sequencing with primers seq 421L and seq 422L (S1 Table) in the results of the batchwise screening.

The results showed that the sensitivity of the batchwise screening will depend on both the variant allele and the primer used for sequencing (S2 Fig.). Furthermore, a low contribution of a variant in the mixture cannot always be distinguished from background noise, limiting the detection of variants only present on few KIV-2 copies within a sample. The detection threshold varied greatly and ranged from 5% to 30%, also depending on the sequence quality. Above the detection level, the signal intensity of a variant correlated with the proportion of the variant in the sample. However, in general sequence context-dependent incorporation of dideoxynucleotides [20] can result in the proportions of peak heights in the electropherogram not to correlate linearly with the actual ratio of variant base carrying CNV units [21], in our case KIV-2 repeat units.

### Quality check for intra-allelic frequencies

To control for any bias in the estimated intra-allelic variant frequencies which could have been caused by stochastic effects during PCR amplification or cycle sequencing [21], we performed the same PCR (either 421 or 422) several times for particular samples and sequenced the PCR products with the same primers as before in the original batchwise screening. Three different samples each for the splice site mutation, and for type B specific variants, were used for this evaluation. Only negligible differences in the intra-allelic frequency estimates were observed for all the sequencing reactions conducted, both for comparisons between reactions from the same day, as for those between reactions of different days (S3 Fig.).

### Quality check for allele dropout

In order to check for possible allele dropout due to unknown sequence variation in the primer annealing sites for the PCR fragments 421 and 422, we designed two alternate PCRs. Their amplicons contained both exons; one (fragment 421to422, 5105 bp) with the long intron in between exon 1 and exon 2 of the same KIV-2 unit, the other (fragment 422to421, 2465 bp) including the short intron between exon 2 of one KIV-2 copy and the exon 1 of the next copy. While the 422to421 PCR used a combination of primers from the PCRs for 421 and 422 (Fig. 3), 421to422 PCR used the other primer of the PCR 422 with a new lower primer, as the corresponding lower primer of the 421 PCR proved to be incompatible due to strong dimer formation (S1 and S2 Tables). Since the intron sequences flanking the KIV-2 CNV diverge from those within the CNV, in any allele both the first KIV-2 exon 1, located downstream of KIV-1 exon 2, and the last KIV-2 exon 2, located upstream of KIV-3 exon 1, would not have been amplified by the 422to421 PCR.

These PCRs were amplified using the Long range PCR kit from Qiagen (S2 Table). After cleaning up with ExoSAP-IT enzyme system (Affymetrix), the PCR products were used for cycle sequencing and subsequent sequence analysis. We submitted 10% of alleles, representing the array of populations, to this additional screening. For the corresponding regions, no other variable sites than those observed in the amplicons 421 and 422 were discovered by these comparisons.

### Nomenclature of variants

The human reference sequence for *LPA* (ENSG00000198670.7; Chromosome 6: 160,952,515–161,087,407 reverse strand; GRCh37) contains 6 KIV-2 copies, which we count by roman numerals (S4 and S5 Tables). The exons of these copies are located at g.19209 to g.19368, g.23367 to g.23548, g.24756 to g.24915, g.28911 to g.29092, g.30303 to g.30462, g.34460 to g.34641, g.35849 to g.36008, g.40004 to g.40185, g.41393 to g.41552, g.45550 to g.45731, g.46939 to g.47098, and g.51102 to g.51283, always alternating a 160 bp long first and a 182 bp long second exon of the KIV-2 domains, and cover c.392 to c.2443 of *LPA*.

Our approach did not allow assigning any variants to specific KIV-2 copies in the alleles. Furthermore, the actual number of KIV-2 copies ranges from 1 to >40 in individual alleles. Thus allocating any variants within the KIV-2 CNV to a genomic position in relation to the reference sequence is not possible. Additionally, we report several new variants, to which no rs number has been assigned yet. However, even for sequence variants already described within the KIV-2 CNV, different rs numbers have been assigned to variants located at the same exonic positions. Without a technique to properly determine the actual physical order of individual KIV-2 copies in a given *LPA* allele, any variation could be present on any of the KIV-2 copies – or on several of them – and accordingly several genomic positions could be assigned to them.

Consequently, we will give the positions of all variants according to the respective exons, i.e. either KIV-2 exon 1 (K421) or KIV-2 exon 2 (K422) throughout our results and discussion. Additionally, in S4 and S5 Tables, we have listed the chromosomal positions for each variant according to the first KIV-2 copy of *LPA*. We give all bases according to the reverse strand, which is the coding strand for *LPA*.

## Results

### Cloning of genomic KIV-2 copies from a Bantu individual

We cloned fragments 421 and 422 (see Material and Methods and Fig. 3, S1 Table), containing KIV-2 exon 1 or KIV-2 exon 2 respectively, from genomic DNA of a South African Bantu (sample ID S1) with a KIV-CNV genotype of 18 and 32 KIV copies on the short and long *LPA* alleles. These fragments were then subjected to Sanger-sequencing of the exonic regions and the directly flanking intron sequences with a coverage of 2.7x for 421, and 3.2x for 422. No sequence variation was observed in the exonic region and the immediate flanking intron sequence of KIV-2 exon 1 (K421) in any of the clones. For the second exon (K422), two synonymous variants, A>T at position 31 (K422 31A>T, for nomenclature of variants see Materials and Methods) and T>C at position 58 (K422 58T>C), were detected in 9 of the clones each, but never on the same clones (Table 1).

**Table 1:** Comparison of cloning results with batchwise sequencing of the same sample.

Method	Samples	Variable site in KIV-2 exon 2	
		K422 31A>T	K422 58T>C
	Reference Sequence	A	T
**Cloning**	141 /159 clones	A	T
	9 /159 clones	A	C
	9 /159 clones	T	T
**Batchwise screening**	Genomic DNA	A	T
	Short allele	A	95% T; 5% C
	Long allele	A	T

By sequencing clones of amplicons 421 and 422 from the genomic DNA of a South African Bantu (ID S1), two variants were found in KIV-2 exon 2, both in nine clones. They are located at bases 31 and 58 of the exon. Neither of these variants could be detected in the batchwise sequencing of the genomic DNA of the same sample, which comprised 50 KIV-2 copies (18 on the short (S1 I) and 32 on the long allele (S1 II)). In the batchwise screening conducted in the separated alleles, only the variant K422 58T>C appeared slightly visible on the short allele of the sample.

### Batchwise analysis of genomic DNA from the Bantu individual

The same regions amplified from the same genomic DNA which we had used for the cloning were screened in a batch-wise approach. Following the simultaneous amplification and sequencing of all 50 KIV-2 copies in one reaction, the obtained sequence appeared to be identical to the reference sequence of the region at all positions upon visual analysis of the electropherogram in the sequence analysis program. From the cloning results, we expected approximately 6% of the KIV-2 repeats to harbor one of the two exonic variants (Table 1). However, even in a good quality Sanger sequence, this might be below the detection threshold. This was further investigated by dilution experiments using the previously sequenced clones (see Material and Methods and S2 Fig.). We also conducted some experiments to control for possible allelic dropout of our PCRs and for the reproducibility of sequencing results (see Material and Methods) and found no indication for either issue biasing our results.

### Batchwise sequence analysis of 90 separated alleles

To increase the sensitivity in the batch-wise screening, we used DNA from separated alleles instead of genomic samples (S1 Fig.). This lowers the number of KIV-2 copies in any batchwise PCR product and also allows to study directly the association of any sequence variants, if found, with KIV-2 allele size (Fig. 2, panels F and G). By this batch-wise sequencing, we screened 90 alleles separated by PFGE from 45 individuals of six different populations (10 Hong Kong Chinese, Austrians, and Gabonese Bantu each, 5 Egyptians, South African Bantu, and Khoi San each, hence 40% of Sub-Saharan and non-African origin each, and 10% of North African origin, for details see Materials and Methods). PCR fragments 421 and 422 (Fig. 3), were amplified and subjected to sequencing, using the primers specified in S1 Table.

**Fig 4:**
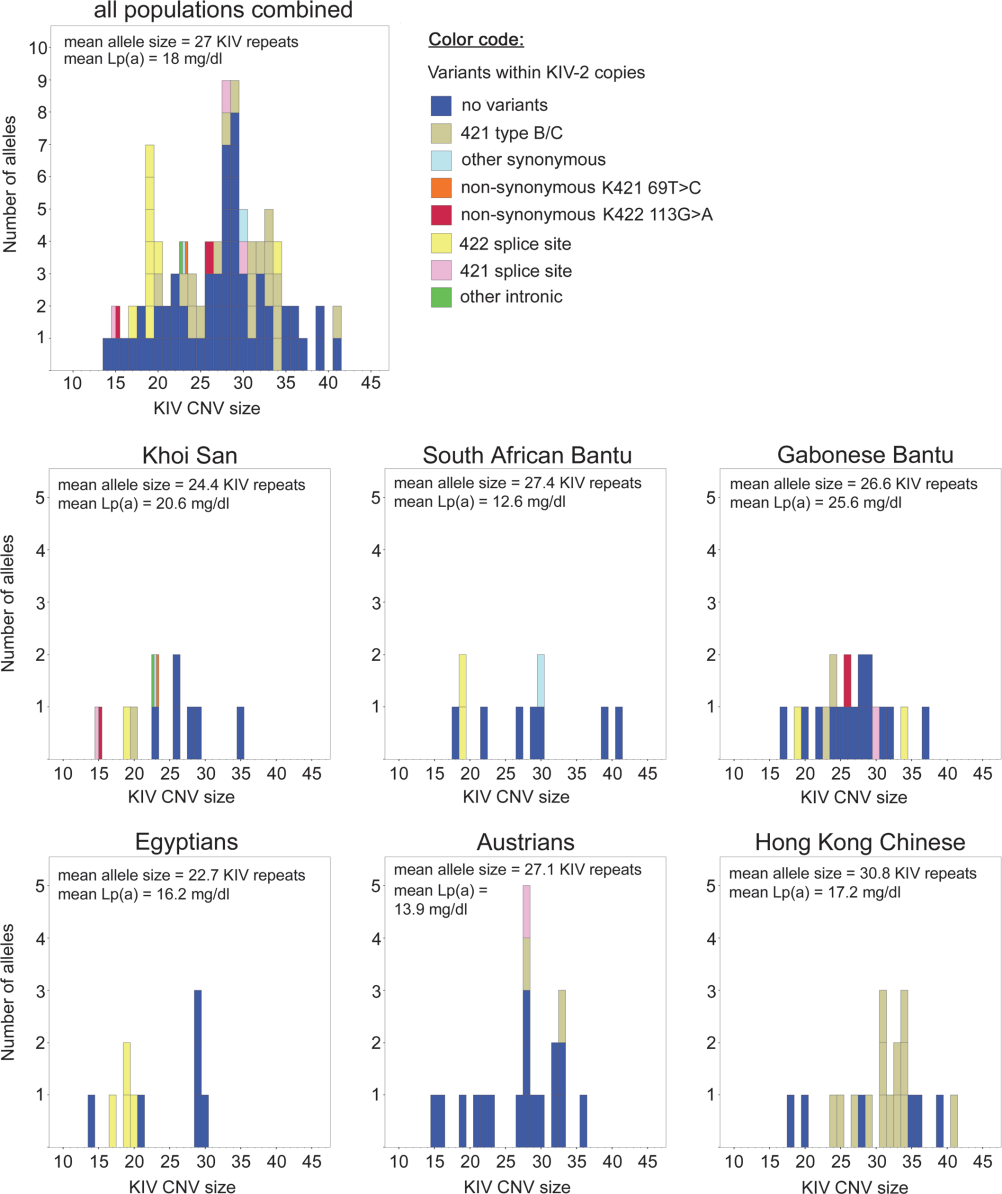
Distribution of sequence variation within KIV-2 across KIV CNV alleles and populations. The frequency distribution of the KIV CNV alleles is given for all samples, and by population. Alleles carrying variants within the screened KIV-2 exon 1 and KIV-2 exon 2 domains are colored according to the variation found in the batchwise screening. Alleles harboring several variants are striped. Note that for the individual alleles, the number of KIV-2 copies carrying the depicted variants cannot be derived from this figure. See S6 Table for these intra-allelic frequencies. Inserts show the mean KIV CNV sizes (i.e. number of KIV-2 repeats plus the nine non-repetitive KIVs), and the mean allele-associated Lp(a) concentrations in mg/dl for the populations.

Only two alleles, both from a Khoi San, showed variation in both the first and second exon of the KIV-2 simultaneously (Fig. 4; S6 Table). 56 of the 90 alleles did not display any clearly detectable variation at all. For the fragment 421, most of the variant carrying alleles were from non-Africans (17 versus 7), and 42% of all non-Africans but only 20% of the African alleles, all of them from Sub-Saharan Africa, carried variation in this region. For fragment 422, all 12 variant carrying alleles were from Africans, and here the frequency was higher in the North Africans from Egypt (40%) than in the Sub-Saharan Africans (20%).

Despite the higher rate of clearly detectable variants in the region of KIV-2 exon 1 in non-Africans, all non-synonymous variation was found in Africans. In both the first and second exon of KIV-2, only one non-synonymous variant was detected (Tables 2 and 3). The variant at position 69 in the first exon (K421 69T>C) was observed in an allele from a Khoi San and leads to a change from Tyr to His in the affected domain (p.Tyr154His; Table 2, S4 Fig.). The intra-allelic frequency (i.e. the proportion of KIV-2 copies carrying the variant within the allele) was estimated at 10% (S6 Table). Since the respective allele carries 14 KIV-2 copies, one or two copies are expected to harbor this variant. However, as was shown by the dilution experiment (S2 Fig.), these proportions might correspond only roughly to actual repeat numbers carrying the variant. The non-synonymous variant in the second exon, K422 113G>A (Table 3, S4 Fig.), introduces an Ala to Thr substitution (p.Ala222Thr). It was found on two African alleles (Gabonese and Khoi San), with an intra-allelic frequency estimated at 10% and 15%, respectively (Table 3, S6 Table). As the KIV-2 CNV size of the Khoi San allele was very short (6 repeats), this likely corresponds to a single KIV-2 copy carrying this variant.

**Table 2:** Sequence variation in fragment 421.

		Position according to exon (K421)																	
		4	14	20	41	53	69	71	86	92	103	113	133	+1	+6	+12	+41	+54	+99
**Ref. sequence**	**K421 A**	C	A	T	T	G	T	C	A	A	C	T	G	G	T	T	G	T	A
	**K421 B**		G		C				T										
	**K421 C**		G		C														
**Ensembl**	**K421 I**	Y							M		Y					Y			
	**K421 II**								M		Y					Y			
	**K421 III**				Y	R		Y	H				K		W	Y	S	K	W
	**K421 IV**								M		Y					Y			
	**K421 V**								M	R	Y					Y			
	**K421 VI**								M		Y					Y			
**Own data**	**K421**		R	Y	Y		Y		W			Y		R					
	**SNP ID**		K421 14A>G	K421 20T>C	K421 41T>C		K421 69T>C*		K421 86A>T			K421113T>C		K421 +1G>A					
	**Intra-allelic MAF**		5% to 30%	15%	5% to 45%		10%		5% to 30%			15%		5% to 15%					
	**Populations**		K; G; A; H	K	K; G; A; H				G; A; H			S		K, G, A					

K: Khoi San; G: Gabonese; S: South Africans; A: Austrians; H: Chinese;

*: non-synonymous variant Tyr→His (p.Tyr154His)

**Table 3:** Sequence variation in fragment 422.

		Position according to exon (K422)														
		-28	-17	-6	27	58	101	113	150	155	160	170	171	+15	+40	-28
**Ref. sequence**		G	C	T	A	T	C	G	C	C	C	C	C	G	T	G
**Ensembl**	**K422 I**	K	S		R					Y						K
	**K422 II**					Y	S	R	Y		M	Y	Y	K	W	
	**K422 III**															
	**K422 IV**															
	**K422 V**								Y							
	**K422 VI**															
**Own data**	**K422**		S	K				R								
	**SNP ID**		K422 -17C>G	K422 -6T>G				K422 113G>A*								
	**Intra-allelic MAF**		30%	30% to 50%				10% to 15%								
	**Populations**		K	K; G; S; E				K; G								

K: Khoi San; G: Gabonese; S: South Africans; E: Egyptians;

*: non-synonymous variant Ala →Thr (p.Ala222Thr)

All sequence variation allocated to KIV-2 exon 1 (K421) domains and their flanking introns (bases -15 to +100) in the Ensembl sequence (ENSG00000198670; GRCh37, integrating the 1000 Genomes Project release 16) is compared with the own results from the batchwise screening of the same region. Positions are given according to the exon; IUPAC nucleotide codes are for the reverse strand. KIV-2 exon 1 (K421) reference sequences are given for K421 type A, B, and C. In the human reference genome, the third of the six KIV-2 copies (K421 III) is of type B, all other five are of type A. Positions 14, 41, and 86 distinguish between these different types. For a critical evaluation of the variation reported by the databases for position 86, see S5 Table. For our own results, we do not give allocation to specific K421 copies, since their number varies depending on the size of the individual alleles. Instead, we give the intra-allelic frequencies (“intra-allelic MAF”). If a variant was found on different individual alleles, we give the range of these frequencies. The ratios of wild type to variant alleles carrying KIV-2 copies were estimated by the relative height of the two peaks in the electropherogram. The populations in which the variants were observed are indicated. For individual results see S6 Table (own data) and S4 Table (external data).

All sequence variation allocated to KIV-2 exon 2 (K422) domains and their flanking introns (bases -30 to +40) in the Ensembl sequence (ENSG00000198670; GRCh37, integrating the 1000 Genomes Project release 16) is compared with the own results from the batchwise screening of the same region. Positions are given according to the exon; IUPAC nucleotide codes are for the reverse strand. For our own results, we do not give allocation to specific K422 copies, since their number varies depending on the size of the individual alleles. Instead, we give the intra-allelic frequencies (“intra-allelic MAF”). If a variant was found on different individual alleles, we give the range of these frequencies. The ratios of wild type to variant alleles carrying KIV-2 copies were estimated by the relative height of the two peaks in the electropherogram. The populations in which the variants were observed are indicated. For individual results see S6 Table (own data) and S5 Table (external data).

**Fig 5:**
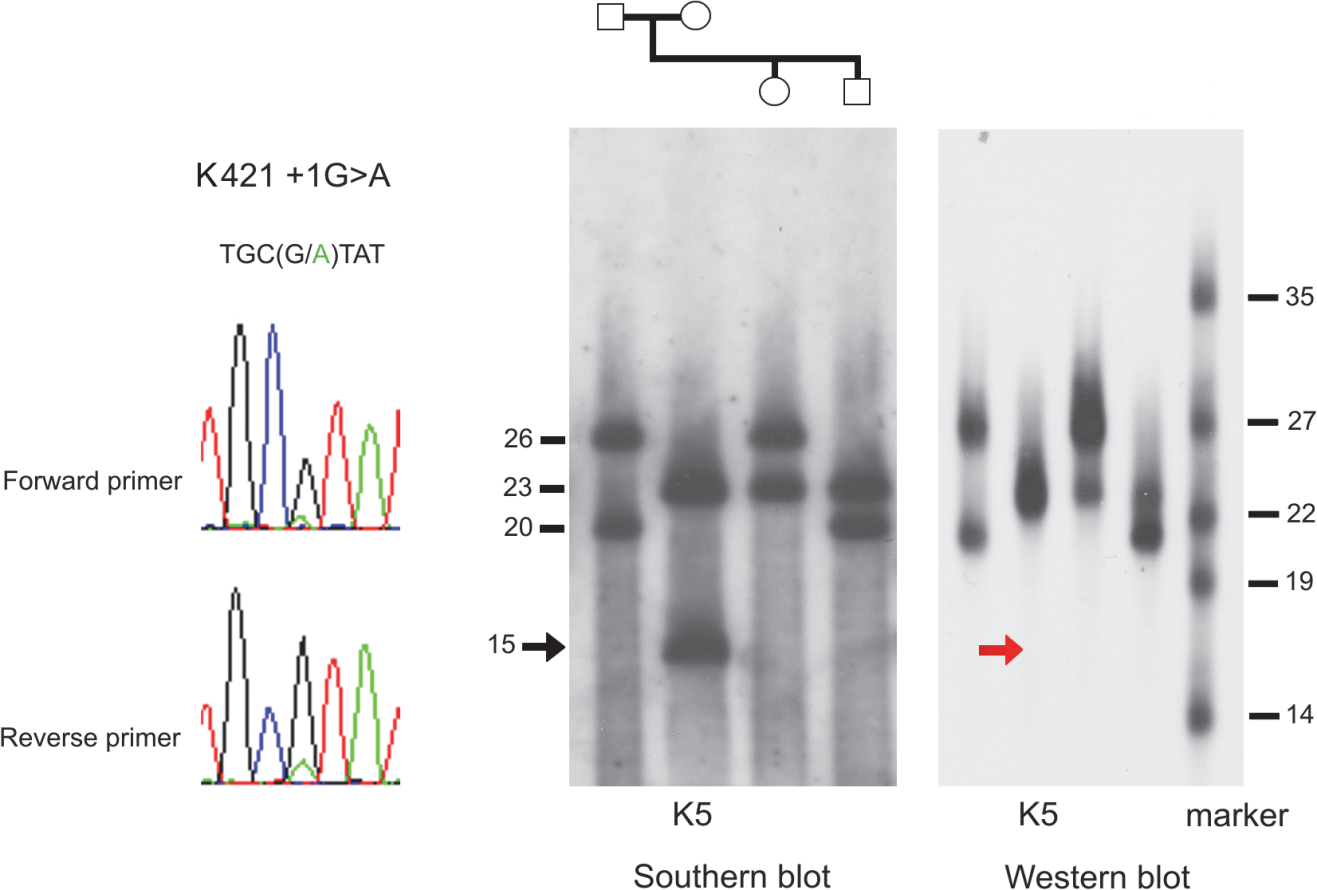
Donor splice site K421 +1G>A and its association with a null allele. Southern and Western blots for the Khoi San sample (K5) harboring the donor splice site mutation K421 +1G>A on its short allele with 15 KIV repeats are shown in context with family members of the individual (family members are not included in the analyses of the present study). The electropherogram from the batchwise sequencing for this allele is shown on the left. KIV CNV sizes for the Southern Blot are given for all family members, and for the Western blot the sizes of the apo(a) isoforms size marker is shown on the left. While both alleles of the sample K5 are clearly visible in the Southern blot (15 and 23 KIV copies), the corresponding isoform for the short allele is missing in the Western blot, indicating a direct effect of the donor splice site mutation on the phenotype by introducing a null allele. Note that this Western Blot is exposed for a long time, so as not to miss low expressed isoforms.

The same short Khoi San allele also harbored a donor splice site G>A mutation at position +1 in K421 (Table 2, Fig. 5, S6 Table), again at a frequency estimated at 15% and thus most likely affecting one KIV-2 copy only. The same variant was also present on one allele of a Gabonese Bantu and one allele in the sample from Austria (Fig. 4; S6 Table). However, the signal intensity for the variant allele was at the lower border of the detection threshold in these latter two samples. Both of these alleles were long (21 and 19 KIV-2 repeats, respectively), and the estimated intra-allelic frequency of 5% would again correspond to a single KIV-2 copy carrying the splice site mutation.

A second variant in the flanking intron regions was found on several alleles, but only in the four African populations, with population frequencies ranging from 10% to 40% and being highest in the Egyptians (Fig. 4, S6 Table). This putative acceptor splice site mutation in the 6th base upstream of the second exon (K422 -6T>G, S4 Fig.), interrupts the semi-conserved acceptor splice site’s poly-pyrimidine sequence and might affect mRNA splicing. It was observed mainly on short (8 to 11 KIV-2 repeats), but also on a much longer KIV-2 allele (25 KIV-2 repeats), always with intra-allelic frequencies ranging from 30% to 50% (Table 3, S6 Table).

Concerning synonymous sequence variants, the second exon appeared to be more conserved than the first one, as none was detected in the former. In total, synonymous variants were found at five positions in the first exon (S4 Fig., Table 2). Two of them, K421 20T>C and K421 113T>C were rare, as they were restricted to one single allele in a Khoi San and a South African sample respectively. Both of these variants were estimated at 15% intra-allelic frequency. For the South African allele with 21 KIV-2 units, this intra-allelic frequency would correspond to 3 repeats carrying the variant. For the Khoi San allele, the observed intra allelic frequency of 15% would correspond to 2 of its 14 KIV-2 carrying the variant allele of K421 20T>C (S6 Table).

Apart from the two splice site variants, in the 185 bp of flanking intronic sequence which were screened at unambiguous quality for all samples in this study, only one further variant was captured, again in a Khoi San allele. This variant allele of K422 -17C>G was also found at an intra-allelic frequency strongly suggesting it to be present in several KIV-2 copies of the affected allele (S6 Table). Hence also variants with low population frequency appear to be present on several KIV-2 copies of alleles carrying them.

Neither of these variants was detected in specific amplicons from the two KIV-2 flanking regions, KIV-1 exon 2 and KIV-3 exon 1, of the respective samples. At the same time, other variants were found in these two domains on some alleles, including short ones, but did not show up in the batchwise screening of their KIV-2 region. Consequently, we are positive about the correct allocation of the variants found in our batchwise screening to the KIV-2 CNV itself and the specificity of our PCRs.

Three other synonymous variants detected in the 421 amplicons are much more frequent, both in terms of the population and their intra-allelic frequencies. These three variants differentiate between the so-called KIV-2 type A, B or C. While detected in all three major population groups (Africans, Europeans, and Asians), they were by far most common in the Hong Kong Chinese (Fig. 4, S6 Table), reaching an allele frequency of 50% for type B, and 20% for type C. In fact, these variants were the only ones clearly detectable by the batchwise screening in non-African alleles.

Since only the variable site K421 86A>T differentiates between type B and C (with type B carrying the variant allele), a simultaneous presence of both types on some alleles cannot be excluded, given the margin of error in the estimation of intra-allelic frequencies. For the same reason, some alleles identified as carrying solely type C could indeed carry also type B, as these samples typically showed low intra-allelic variant allele frequencies (S6 Table). Most of the type B carrying alleles, especially in the Chinese, however, showed a high intra-allelic frequency for all three variants, and this frequency was similar for several alleles covering a wide range of KIV-2 CNV sizes.

The two synonymous variants which were found in 6% of the clones of the South African sample S1 each could not be unambiguously identified in the batchwise approach from separated alleles (Table 1). Only for the short allele with 18 KIV-2, the variant base of K422 58T>C was visible, but would have been missed without previous information from the cloning that this position was expected to harbor a variable site.

### Phenotypic relevance of the variants

With the restricted sample size for each population in our study, a meaningful statistical evaluation for any association of variants with Lp(a) concentrations is not possible. This notwithstanding, for some samples estimates of allele associated Lp(a) concentrations were available from the semi quantitative evaluation of Western Blots. This included the Khoi San sample with 6 KIV-2 repeats carrying the donor splice site variant K421 +1G>A (Fig. 4, S6 Table). This allele appeared as a phenotypic null allele, i.e. allele with no corresponding apo(a) isoform in plasma (Fig. 5). The non-synonymous variant K422 113G>A, found on the same Khoi San allele, is also present in a Gabonese allele, where it is not associated with a null allele (S6 Table). In contrast, the two other alleles suspected to harbor the donor splice site variant were associated with no or very low allele associated Lp(a) (S6 Table). However, while the absence of any detectable apo(a) isoforms is remarkable for a short allele, for longer alleles low Lp(a) concentrations are not out of the ordinary. The putative acceptor splice site mutation K422 -6T>G was not associated with a null allele.

## Discussion

The analysis of sequence variation within regions of the genome harboring complex or multiallelic CNVs is a challenge even with present day deep sequencing methods [22, 23]. It is therefore not surprising that previous studies of the KIV-2 CNV in *LPA* resulted in controversial conclusions and missed variation of potential functional importance.

Some previous studies [12,13] inferred a high sequence conservation within KIV-2 copies, while another expected much more variation to be revealed in this repetitive region [10]. However, all of these studies were mainly limited to samples of European descent, and based on few [12,13] or even single samples [2,10]. The now accessible data from large genome resequencing projects like the 1000 Genomes Project (1000G) also report only comparatively few and mostly low frequency variants for the exonic regions of KIV-2. In total, only eight and nine different positions each are listed as variable within the first and second exons of KIV-2 respectively in these databases (see ensemble ENSG00000198670, GRCh37). Several of these variants were allocated to specific KIV-2 units of the reference sequence only (Tables 2 and 3, S4 and S5 Tables).

The results from our focused, systematic resequencing which took into account peculiarities of the *LPA* locus question the high degree of sequence conservation inside this CNV, at least for Africans

### Batchwise screening

From the 10 different variants observed in the African samples, neither the synonymous variation K421 113T>C in exon 1 nor both of the splice site mutations, K422 -6T>G and K421 +1G>A have been reported by any other study before. In contrast, we did not find any new variants in the 20 alleles from the Hong Kong Chinese screened by the same method. In the Austrians only the donor splice site variant K421 +1G>A was observed as a new variant, but would not have been identified if it had not been clearly seen at a higher intra-allelic frequency in the Khoi San, thus already marking it as a variable site.

Despite the slight difference in sample size our results point towards a higher degree of sequence variation within the KIV-2 CNV in Africans. This is in line with expectations from other genomic regions [24], and the other *LPA* domains [25]. However, the sample size of 90 alleles is too small to draw final conclusions. Additionally, limitations of the available screening techniques have to be taken into account. Due to the exceptionally high sequence homology separate amplification of individual KIV-2 units is impossible. With the approach of batchwise sequencing, all the KIV-2 copies in a sample are expected to be amplified simultaneously, and any position in the sequence can only be analyzed for the cumulative bases of all units amplified. This sets limits to the sensitivity of the method for the detection of sequence variation, even when reducing the number of KIV-2 repeats in any batch by analyses of separated alleles instead of diploid sample DNA, as we did in our study.

### Cloning

In contrast, cloning provides direct access to single KIV-2 units and hence offers the ability to detect low frequency variants. Indeed, by cloning, two synonymous variants were found which were not unambiguously detectable in the batchwise analysis of the same sample. One of these variable sites, K422 31A>T, has not been described before. In the previous studies however, cloning results have reported an abundance of sequence variation, many of them non-synonymous but mostly present only in single clones [10,13] (S4 and S5 Tables). Our results stand out as we found both of the variants on 9 clones each, indicating a high intra-allelic frequency, whereas no other exonic variation was detected. We consider it unlikely to have missed much of the variation actually present in the sample. With the number of clones analyzed, the probability to observe any variation present on a single KIV-2 repeat of the sample in at least one clone was 93% for amplicon 421 and 96% for amplicon 422.

The extent and type of variation reported in the previous cloning studies has to be viewed with caution. The cloning results from four alleles of two Norwegian individuals [13] were biased against KIV-2 exon 1 type B and type C units, as one of the primers used in that study was specific for type A units [13]. At the same time, primer specificity to distinguish KIV-2 from other KIV units was questionable, the coverage was low, and the enzyme used had a lower accuracy [26], which altogether might have enhanced the number of artifacts in that study. The authors of the study conducted on the cloning of another European allele had already speculated themselves that many variants were artifacts, as they were only found on one clone each [10].

### Sensitivity of the screening method

While cloning might be ideal to detect the full range of sequence variation in the KIV-2 CNV of an individual if properly applied, its application is limited by the number of samples which can be processed. On the other hand the batchwise sequencing must be expected to underestimate variation. The signal intensity of variant bases in the electropherogram can be below a detection threshold when a variant is only found in a small proportion of KIV-2 copies within an allele (Fig. 2). As we estimated in our dilution experiment (S2 Fig.), this threshold varies from 5% to 30%.

These limiting effects on the detection level will be more pronounced for long alleles, while any variation is more likely to be detected if present on a short allele, as was illustrated by the donor splice site K421 +1G>A. Alleles with up to 10 KIV-2 units make up only 16.7% of our sample, but represent 53% of variant carrying alleles when excluding the K421 type B and C defining variants. In such short alleles, even one single variant carrying KIV-2 unit would equal an intra-allelic frequency of ≥10%, which does enhance the chance to pass the detection threshold in the batchwise screening approach (Fig. 2C). In general, East and Southeast Asian populations have a higher frequency of long KIV-2 CNV alleles than Europeans and especially Africans ([27], Fig. 4, S6 Table). 22% of all African alleles, but only 10% of the non-Africans alleles analyzed in our study were in this size range of up to 10 KIV-2 repeats. Consequently, our finding of more sequence variants in the African samples might reflect such bias. Likewise, the absence of sequence variation reported for this genomic regions in the East Asian samples of the 1000G panel could be due to a similar effect of KIV-2 CNV size on detection thresholds, e.g. by the error margins set in the evaluation of short reads in next generation sequencing (NGS), especially when still at low coverage [22,24].

Nevertheless it is notable that in our study apart from the K421 B and C defining variants and the donor splice site mutation, all variation was only found in African alleles, especially since some of these variants were also detected on longer alleles. Moreover, some variant bases, especially the putative acceptor splice site variant K422 -6T>G but also the intronic variation K422 -17C>G, were estimated to be at intra-allelic frequencies which indicate that they must be present on more than one KIV-2 copy in individual *LPA* alleles (Fig. 2). Hence, the detection bias conferred by allele size likely explains only some of the observed differences in variant frequencies between populations.

### Distribution of variants

A likely explanation for the paucity of sequence variation in the non-African populations is population history in combination with the unique features of the KIV-2 CNV. As outlined above, point mutations affecting only one KIV-2 copy will likely be missed in the batchwise screening unless they occur on short KIV-2 alleles (Fig. 2C). As a consequence, young variants which arose after the split of the different populations are less likely to be found, especially in populations with a high frequency of long KIV-2 alleles. Spreading of variants across the KIV-2 CNV, leading to (some) alleles with high intra-allelic variant frequencies, is expected to correlate with the age of a variant. This might result in a detection bias in favor of older variants and could also explain why in the 1000G or the ESP panel most variants, even when at low population frequency, are shared between European and African populations (Fig. 4, S4 and S5 Tables), while the majority of rare variants in the genome appears not to be shared between diverged populations [28].

In our much smaller data set, only the KIV-2 exon 1 type B defining variants and the putative splice site variation K422 -6T>G were found both at high population and intra-allelic frequencies. It is remarkable that KIV-2 CNV alleles of different size harbor similar intra-allelic frequencies for these variants. Other variant alleles were also detected at intra-allelic frequencies strongly suggesting them to be carried on more than one KIV-2 unit (Tables 2 and 3, and S6 Table). A likely explanation is that non-allelic (interlocus; regarding the paralogous KIV-2 copies as different loci on the same *LPA* allele) gene-conversion [29] spreads variants within KIV-2 CNV alleles. The – possibly interdependent [30] – role and mechanism of CNV size changes in this process remain to be elucidated.

Type B or C containing KIV-2 CNV alleles together make up 50% to 70% of all alleles in our Asian sample, while they were restricted to 5% to 10% in the Africans and Europeans. For the type B variation, the high frequency in a Eastern Asian population is in accordance with a serial founder effect and a strong second genetic bottleneck during the peopling of Asia [31,32]. Their shared presence in Sub-Saharan populations and in Europeans indicates that they are evolutionary old variants. The high frequency of these type B carrying KIV-2 copies in Asians should be considered in the assay design when KIV-2 CNV size determination is performed by qPCR [33].

For the putative acceptor splice site variant, the initial out-of-Africa bottleneck might have restricted it to African populations. Again, its presence in different African populations is suggestive of a rather old age, as the split time between Khoi San and other African populations is estimated as at least 50,000 years [34]. It is also found on alleles of different KIV-2 CNV size, though mainly on shorter ones. This variant might be of functional significance, and hence a shift to high population and intra-allele frequencies by non-neutral mechanisms is an interesting hypothesis and deserves further investigations. Likewise, the population frequencies of the donor splice site variation K421 +1G>A and its contribution to varying frequencies of null alleles between populations [27,35] deserves further study.

### Next generation sequencing and the KIV-2 CNV

Despite the limitations of the screening method employed in our study and with a much smaller sample size, we detected sequence variation within the KIV-2 CNV which was not yet reported by the 1000G (S4 and S5 Tables). Most notably, the putative acceptor splice site variant, found at population frequencies above 10% in our African samples, is not yet reported by that large scale resequencing project for the KIV-2 region. In general, NGS has been shown to capture low frequency variants, even in complicated genomic conditions as in biopsies from cancer or in pooled DNA samples [36]. Furthermore, NGS has become a tool to detected copy number variation itself [37,38]. However, in special cases variation can escape the detection by NGS, as was shown for the GC rich CNV in the *MUC1* gene [23]. In the latter case, this was also attributed to difficulties in the ability to place reads within the copied region to individual repeats. In general, accurate mapping of NGS reads, especially when short, in CNV regions has been identified as a challenge in bioinformatics [22]. This is even aggravated if NGS is based on low coverage, as it is the case in the 1000G’s pilot phase [24]. This challenge certainly also exists for the KIV-2 CNV, where the high sequence homology with the 9 non-repetitive KIV within *LPA*, but possibly also homologies with domains within the pseudogene *LPAL2* (MIM *611682) and *PLG* pose further difficulties for a correct sequence alignment. This problem might be highlighted by the allocation of splice site variants to position -6 of KIV-1 exon 2 (chromosomal position 6:161067433; ss1323156606) and to position +1 of KIV-3 exon 1 (chromosomal position 6:161032593; ss1323156442) of *LPA* in the current 1000G release 16. KIV-1 exon 2 shares 100% sequence homology to the KIV-2 exon 2 units, while KIV-3 exon 1 is 100% homologous to KIV-2 exon 1 type B. When we screened with PCRs specific for those regions flanking the KIV-2 CNV, we did not find either of these variants in the corresponding domains of our samples which carried the splice site variants within the KIV-2 CNV itself. An additional challenge can be expected to derive from the very wide range of KIV-2 repeats numbers, theoretically extending from 1 to >40 in haploid and 2 to >80 in diploid samples.

Targeted deep sequencing, i.e. massive parallel sequencing after specific amplification of the respective KIV-2 CNV regions, should however overcome the above mentioned problems of NGS, while also avoiding the CNV-size bias in the sensitivity of our batchwise approach. Our data provide evidence that such an effort can be expected to capture far more genetic variation than is currently available in the databases. If performed on separated *LPA* alleles, this will also shed more light on the evolution of the KIV-2 CNV by allowing for a more extensive analysis on the association of sequence variation within the CNV with CNV size. Furthermore, we report several new variants with effects either on amino acid sequence or possibly splicing and with marked differences in their population frequencies which indicates that further analysis of this gene region can be expected to identify more sequence variations explaining population specific Lp(a) levels [9,10,25,39–41].

## Supporting Information

S1 Fig Allele separation by PFGE for experimental haplotyping of sequence variation within the KIV-2 CNV. Both KIV-2 CNV alleles of all samples had been separated by PFGE before being subjected to the batchwise screening for sequence variation with the KIV-2 CNV. After digestion with kpnI endonuclease, samples were applied twice on the same gel. On the part of gel for cutting the alleles, the samples were loaded in alternate wells (shown as black and grey rectangles respectively) so as to minimize the chances of cross contamination between samples while later cutting the alleles. After PFGE, the gel was cut into two, and the right part was used for detection of the alleles by Southern blotting with a KIV-2 intron specific probe, while the other part was stored in a sealed plastic bag at 4°C. After identifying the position of alleles on the blotting part, the corresponding positions of the alleles on the other part of the gel were excised with a razor blade (shown as white rectangles). Additional slices were cut above and below each main slice and used in case that the main slice did not give positive PCR results, which would have indicated that a slight shift in the position of the bands had occurred during PFGE across the gel resulting in the allele having been missed in the main slice. General homogeneity of the PFGE run was assessed by a molecular size marker visible in the ethidium bromide staining after PFGE (see supplementary online information on Material and Methods). In lane 3 of this blot, the detection did not work at sufficient quality (faint bands not visible in the scan), and separation of the sample was repeated.
(TIF)

S2 Fig Differential dilution experiment to estimate the detection threshold in the batchwise screening. DNA from two clones (A and B) of the amplicon 422 were mixed in different proportions (5:95, 10:90, 20:80, 30:70, 40:60, 50:50, 60:40, 70:30, 80:20, 90:10, and 95:5). The two clones diverged at two positions (SNP1 and SNP2) from each other. The mixtures of these two clones were then subjected to sequencing with the same sequencing primers as used in the batchwise screening. The results indicate a detection threshold in the range of 5% to 30% depending on the sequence quality. Also, the relative heights of the peaks for the two bases depended on the sequencing primers used and the position of the variable site.
(TIF)

S3 Fig Intra-assay quality control for estimated intra-allelic variant frequencies. Electropherograms show results of an intra-assay quality control experiment to assess the fluctuation of estimates for intra-allelic variant frequencies. For the same alleles harboring different variants, the PCR and sequencing was conducted on different days, and on day 2 several times (a, b, c, d, e in panel Day 2) as independent reactions. The fluctuation in the relative peak height of wild type to variant alleles appeared to be negligible for between-day and within-day comparisons.
(TIF)

S4 Fig Electropherograms for samples carrying variants found in the batchwise screening of amplicons 421 and 422. Sequencing results with the forward and reverse primers are shown for variable sites detected in the batchwise screening of the amplicons 421 and 422. The variants at positions 14, 41, and 86 in K421 define the KIV-2 copies of type B or C. In samples harboring KIV-2 exon 1 type B, no clear sequence can be obtained by forward sequencing, as the intron sequence of type A and type B differ by one bp in length. This deletion, which is entered in the reference sequence, is close to the exon, making it impossible to allocate any primer for unaffected forward sequencing of the exonic region.
(TIF)

S1 Protocol Protocols for pulsed field gel electrophoresis (PFGE); Protocol for cloning; Protocol for Cycle Sequencing. (DOC)

S1 Table Primers for PCR and sequencing. PCR and sequencing primer used are given with all their annealing sites according to Homo sapiens chromosome 6, GRCh37.p13 Primary Assembly, which comprises six KIV-2 copies. For PCR primers 421U and 421U_2, seven annealing sites are present, as these primers are also annealing upstream of the non-repetitive KIV-3 exon 1, which is identical to KIV-2 exon 1 type B. However, as the lower primer for the PCR was specific for the KIV-2 region itself, KIV-3 exon 1 was not amplified by the PCR. For primer 422L, there is an additional annealing site outside the KIV-2 CNV downstream of the non-repetitive KIV-1 exon 2, which is identical to KIV-2 exon 2. Here specify of the PCR product is ensured by the KIV-2 specific primer 422U. For the primer combinations, see Fig. 3 and S2 Table. For the sequencing primers, only the annealing sites within the PCR products are shown. No SNPs are reported for any of the primer positions in the reference assembly.
(DOC)

S2 Table PCRs conducted in our study. PCRs for the batchwise screening on DNA from separated alleles demanded 45 cycles, as template concentration after PFGE and cleanup of the cut gel slice was lower than for the genomic sample used for cloning. The batchwise screening of the genomic DNA from the same individual was run at 35 cycles. HOT FIREPol was purchased from Solis BioDyne; Pfu polymerase from Fermentas; and the LR PCR kit from Qiagen.
(DOC)

S3 Table PCRs for flanking regions of the KIV-2 CNV. The specificity of the PCRs for the regions directly flanking the KIV-2 CNV depends on primer 412U in case of 412 (annealing site 6:161,068,509–161,068,527), and on primer 431L (annealing site 6:161,065,900–161,065,923) in case of 431. 421L also anneals in the KIV-2 exon 2 copies, and 431U in KIV-2 domains of type B.
(DOC)

S4 Table Comparison of own results with external data on variants in KIV-2 exon 1. Sequence variation in KIV-2 exon 1 (K421) retrieved from Ensembl (ENSG00000198670; GRCh37) and previous studies in comparison to our findings from the cloning and batchwise screening. The data from external databases are given for populations of African and European descent, for comparison to our study. In the external databases, no variants were reported for Asians. The chromosomal positions shown for variants found in our own study or reported by Rosby et al. (2000) and Parson et al. (2004) are shown only according to the first KIV-2 copy in the reference sequence, as they could be present on any of the KIV-2 copies in the *LPA* alleles screened. Further possible coding positions can be allocated by adding multitudes of 342 bases (as the length of one KIV-2 repeat is 160 bp for exon 1 and 182 bp for exon 2), depending on the length of the KIV-2 CNV in any individual *LPA* allele. KIV-2 exon 1 is labeled as ‘K421’. The Roman numerals (I to VI) with K421 indicate the order of KIV-2 copies in the reference sequence. Chromosomal positions are given as they are entered in the database. Bases are given as on the reverse strand. As the third KIV-2 (K421 III) is the only copy of type B in the reference sequence, sequence reads from KIV-2 type A copies could be entered as variants at the exonic positions 14, 41, and 86 when aligned to the type B copy, and vice versa. Possibly for this latter reason, the frequencies reported by the ClinSeq panel for K421 I, II, IV, V, and VI appear questionable. Additional complication and potential misallocation could also stem from the complete sequence homology between KIV-3 exon 1 and KIV-2 exon 1 type B. A variable site at position 86 of the non-repetitive KIV-3 exon 1 is reported as SNP c.2529T>C (as rs113727842, rs80331546, or rs3798222). Its minor allele frequencies are similar to those reported by the ClinSeq panel for their entries listed above. Hence, we did not include this variation in our discussion.
(DOC)

S5 Table Comparison of own results with external data on variants in KIV-2 exon 2. Sequence variation in KIV-2 exon 2 (K422) retrieved from Ensembl (ENSG00000198670; GRCh37) and previous studies in comparison to our findings from the cloning and batchwise screening. The data from external databases are given for populations of African and European descent, for comparison to our study. In the external databases, no variants were reported for Asians. KIV-2 exon 2 is labeled as ‘K422’. The Roman numerals indicate the order of KIV-2 copies in the reference sequence. For the data retrieved via Ensembl, the chromosomal positions are given as they are entered in the database. In the current build of the reference sequence, any variation reported in Ensemble for K422 is either assigned to the second or fifth KIV-2 copy. The chromosomal positions shown for variants found in our own study or reported by Rosby et al. (2000) and Parson et al. (2004) are shown only according to the first KIV-2 copy in the reference sequence, as they could be present on any of the KIV-2 copies in the *LPA* alleles screened. Further possible coding positions can be allocated by adding multitudes of 342 bases (as the length of one KIV-2 repeat is 160 bp for exon 1 and 182 bp for exon 2), depending on the length of the KIV-2 CNV in any individual *LPA* allele.
(DOC)

S6 Table Variation found by batchwise sequencing of 421 and 422 amplicons. The results from the batchwise screening for the 421 amplicon, containing the first exon of KIV-2 (K421), and the 422 amplicon, containing the second exon of KIV-2 (K422), are entered by population. Alleles are listed with their sample IDs, with Roman numerals representing the short (I) and long (II) alleles of the same sample respectively. Variable sites are named after their position relative to the start of the exon (for the chromosomal positions, see S4 and S5 Tables). For all variants the ratios of wild type to variant alleles carrying KIV-2 copies are given, as estimated by the relative height of the two peaks in the electropherogram. The KIV-CNV allele size is entered as KIV-2 plus the 9 non-repetitive KIV domains. The variable sites at positions 14, 41, and 86 of K421 differentiate KIV-2 exon 1 types A, B, and C, with the wild type allele representing type A. Allele associated Lp(a) concentration are given as derived from densitometric evaluation of Western Blots, where available, and as total Lp(a) of the individual otherwise (indicated by brackets).
(DOC)
