# Supplementary figures and images for: Sequence Variation within the KIV-2 Copy Number Polymorphism of the Human LPA Gene in African, Asian, and European Populations
Source: PLoS One. 2015 Mar 30;10(3):e0121582. doi: 10.1371/journal.pone.0121582 (PMC4378929; doi:10.1371/journal.pone.0121582)

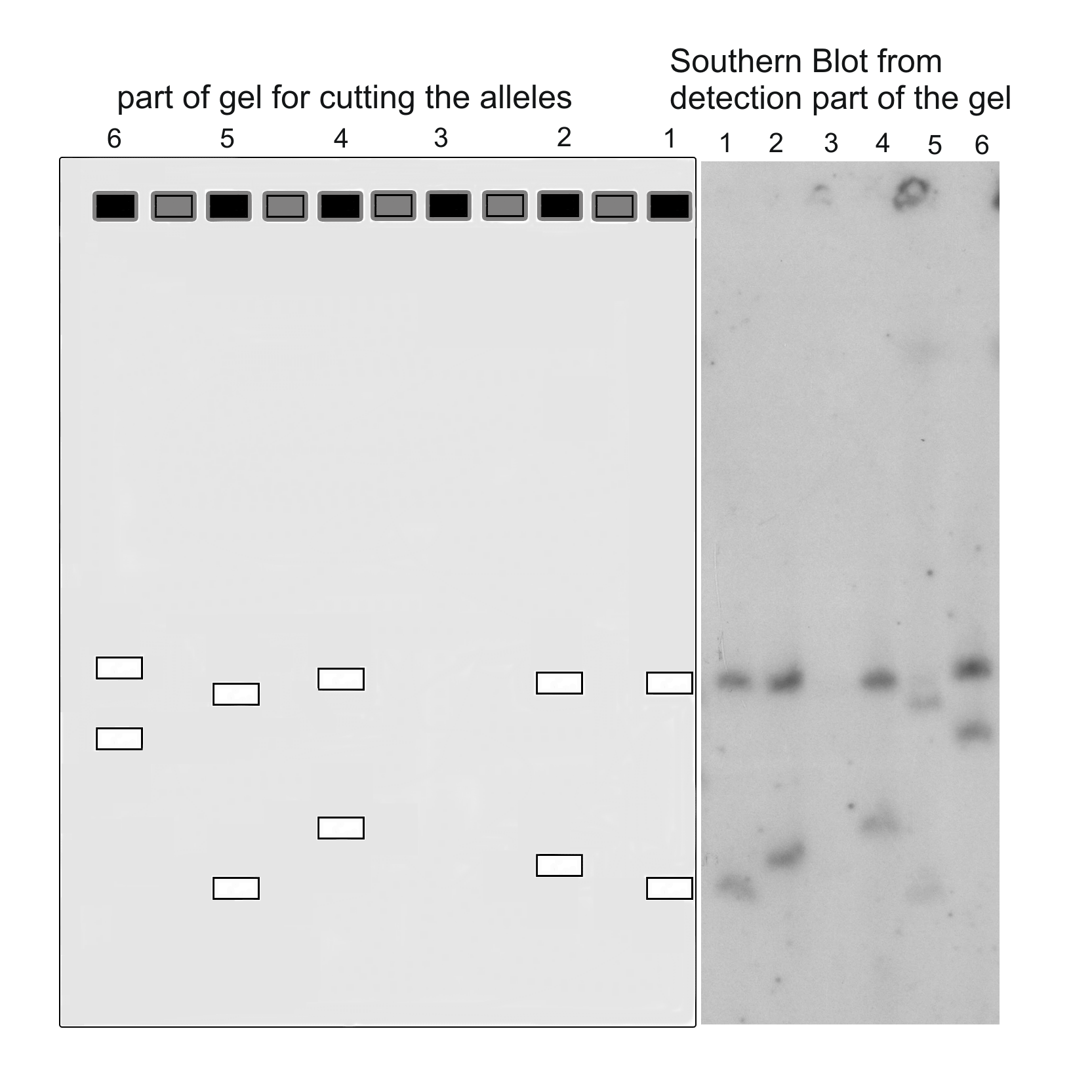

Supplement: S1 Fig — Both KIV-2 CNV alleles of all samples had been separated by PFGE before being subjected to the batchwise screening for sequence variation with the KIV-2 CNV. After digestion with kpnI endonuclease, samples were applied twice on the same gel. On the part of gel for cutting the alleles, the samples were loaded in alternate wells (shown as black and grey rectangles respectively) so as to minimize the chances of cross contamination between samples while later cutting the alleles. After PFGE, the gel was cut into two, and the right part was used for detection of the alleles by Southern blotting with a KIV-2 intron specific probe, while the other part was stored in a sealed plastic bag at 4°C. After identifying the position of alleles on the blotting part, the corresponding positions of the alleles on the other part of the gel were excised with a razor blade (shown as white rectangles). Additional slices were cut above and below each main slice and used in case that the main slice did not give positive PCR results, which would have indicated that a slight shift in the position of the bands had occurred during PFGE across the gel resulting in the allele having been missed in the main slice. General homogeneity of the PFGE run was assessed by a molecular size marker visible in the ethidium bromide staining after PFGE (see supplementary online information on Material and Methods). In lane 3 of this blot, the detection did not work at sufficient quality (faint bands not visible in the scan), and separation of the sample was repeated. (TIF) [file pone.0121582.s001.tif]

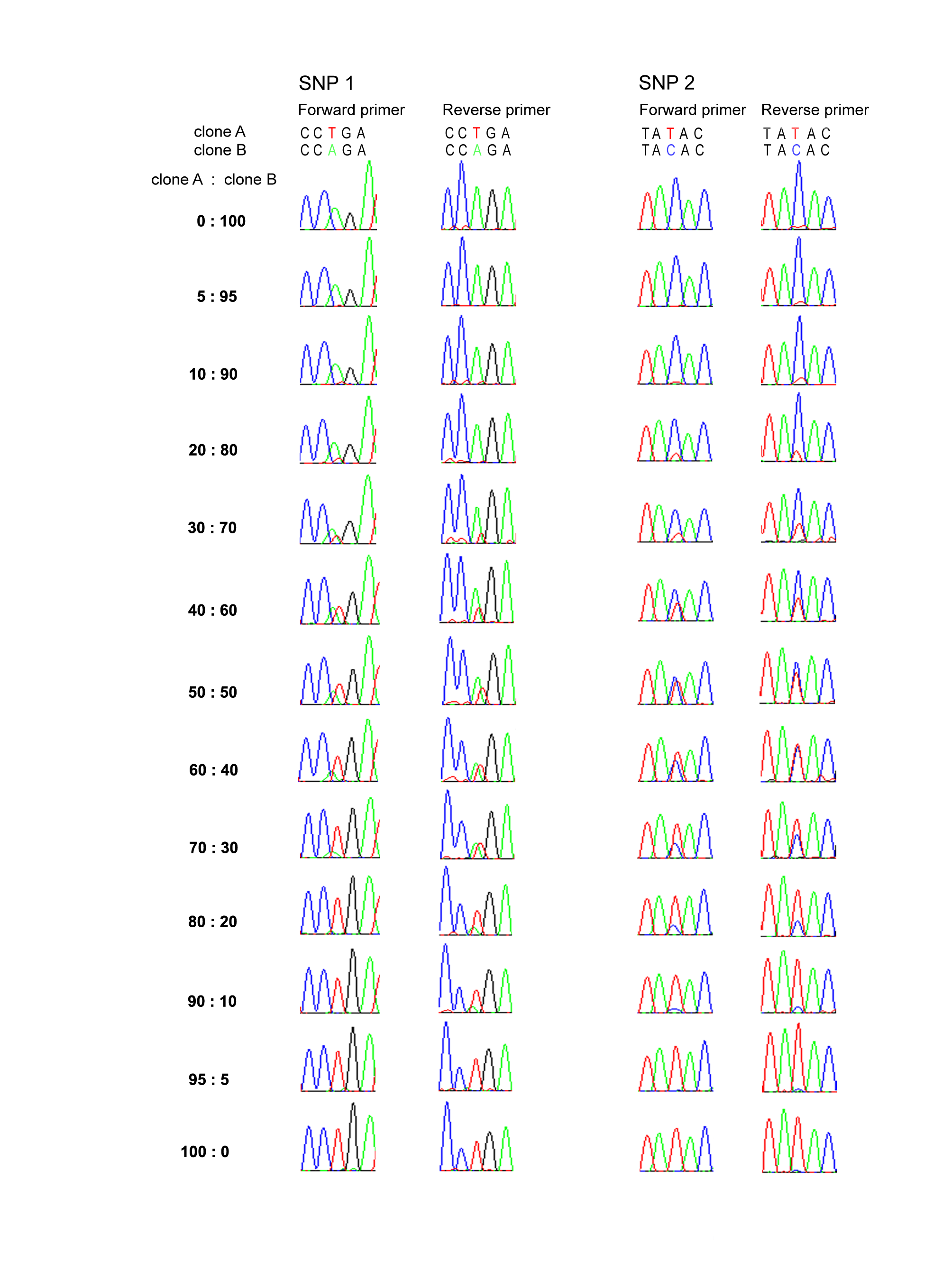

Supplement: S2 Fig — DNA from two clones (A and B) of the amplicon 422 were mixed in different proportions (5:95, 10:90, 20:80, 30:70, 40:60, 50:50, 60:40, 70:30, 80:20, 90:10, and 95:5). The two clones diverged at two positions (SNP1 and SNP2) from each other. The mixtures of these two clones were then subjected to sequencing with the same sequencing primers as used in the batchwise screening. The results indicate a detection threshold in the range of 5% to 30% depending on the sequence quality. Also, the relative heights of the peaks for the two bases depended on the sequencing primers used and the position of the variable site. (TIF) [file pone.0121582.s002.tif]

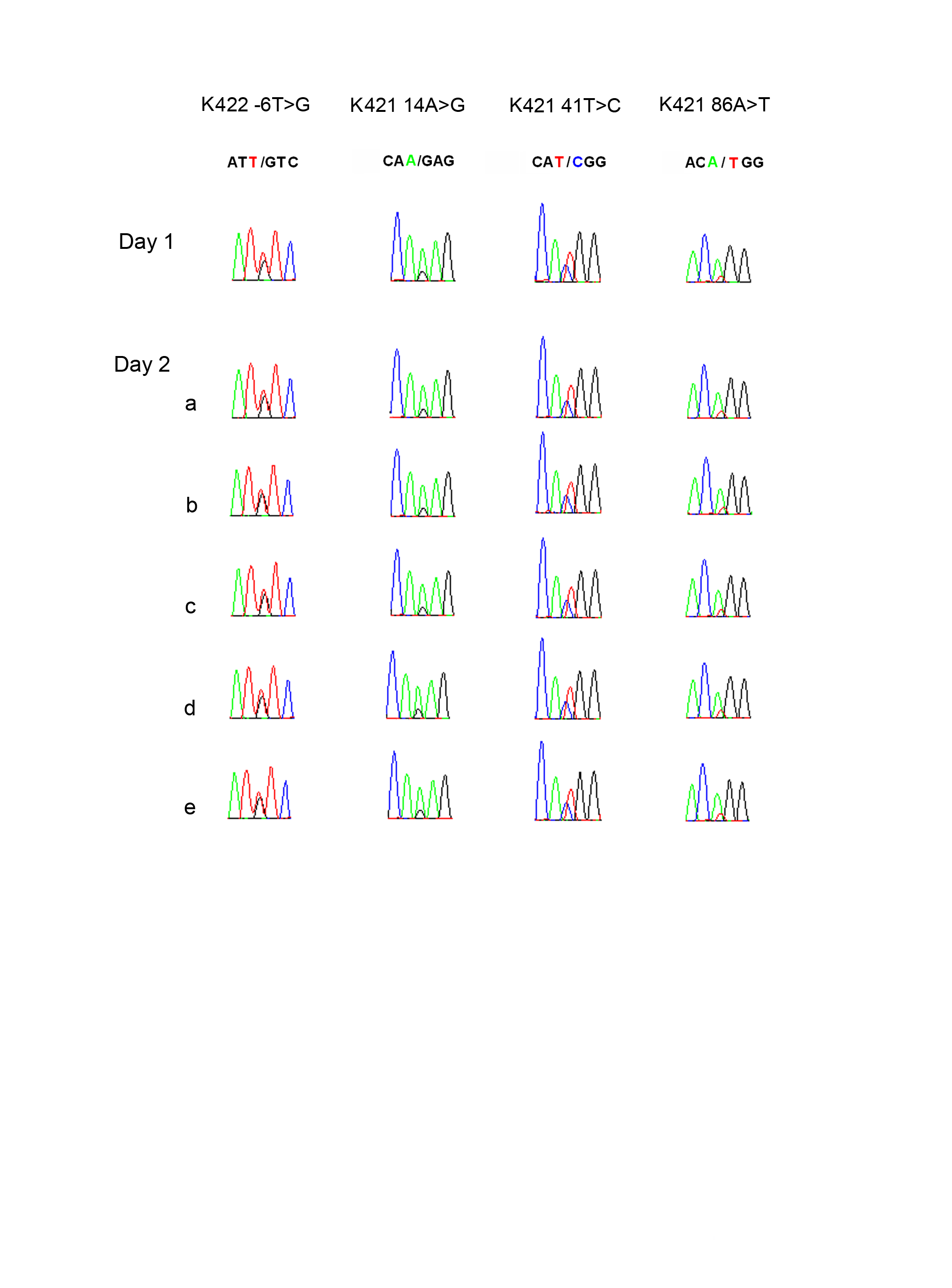

Supplement: S3 Fig — Electropherograms show results of an intra-assay quality control experiment to assess the fluctuation of estimates for intra-allelic variant frequencies. For the same alleles harboring different variants, the PCR and sequencing was conducted on different days, and on day 2 several times (a, b, c, d, e in panel Day 2) as independent reactions. The fluctuation in the relative peak height of wild type to variant alleles appeared to be negligible for between-day and within-day comparisons. (TIF) [file pone.0121582.s003.tif]

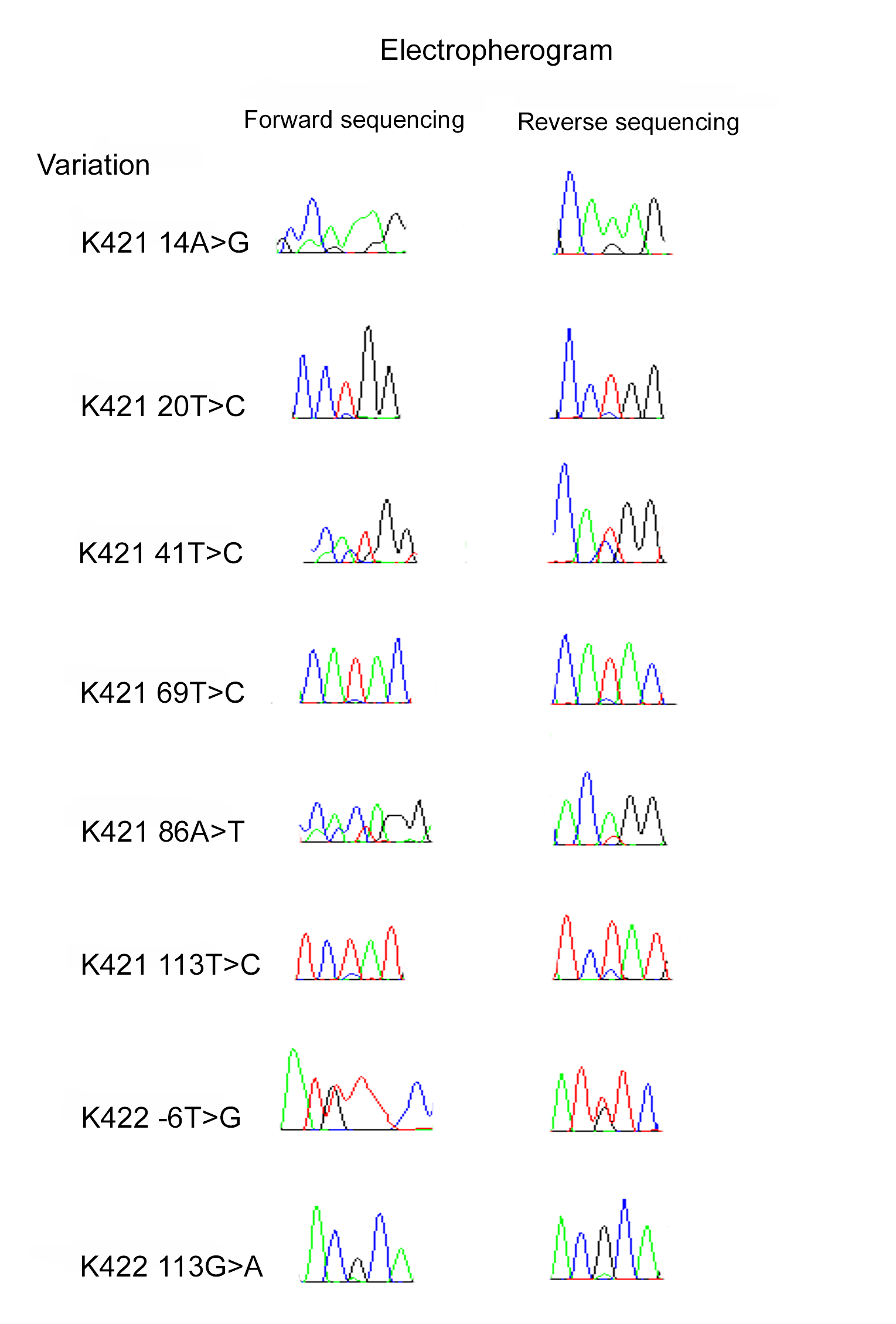

Supplement: S4 Fig — Sequencing results with the forward and reverse primers are shown for variable sites detected in the batchwise screening of the amplicons 421 and 422. The variants at positions 14, 41, and 86 in K421 define the KIV-2 copies of type B or C. In samples harboring KIV-2 exon 1 type B, no clear sequence can be obtained by forward sequencing, as the intron sequence of type A and type B differ by one bp in length. This deletion, which is entered in the reference sequence, is close to the exon, making it impossible to allocate any primer for unaffected forward sequencing of the exonic region. (TIF) [file pone.0121582.s004.tif]
