# Supplementary material for: Sequence Variation within the KIV-2 Copy Number Polymorphism of the Human LPA Gene in African, Asian, and European Populations
Source: PLoS One. 2015 Mar 30;10(3):e0121582. doi: 10.1371/journal.pone.0121582 (PMC4378929; doi:10.1371/journal.pone.0121582)
Supplement: S1 Protocol — (DOC) [file pone.0121582.s005.doc]

S1 Protocol

*Protocols for pulsed field gel electrophoresis (PFGE)*

Half a plug, containing approximately 25µg of DNA within 100 µl volume, was washed 2x for 30 min in 1x TE buffer. Afterwards the plugs were transferred into 1,5 ml Eppendorff tubes and incubated for 2 hours at 37°C with a mix of 5 µl KpnI (MBI Fermentas, 10U/ µl), 20 µl 5 µl KpnI Buffer with BSA (MBI Fermentas) and 125 µl Aqua bidestillata. Incubation was continued for another 2 hours after addition of a further 5 µl KpnI. Tubes were then shortly cooled on ice, and the plugs were then transferred on a 20 cm x 14 cm TAE gel (agarose concentration dependent on the PFGE programme, see below). PFGE was conducted on a Bio-Rad Chef Mapper System in 0.5x TAE. As the possible size range of the KIV-2 CNV containing fragments extends from 34 kb to above 250 kb, we applied the following two different PFGE protocols to optimize size determination.

For cutting the alleles and size determination in samples harboring at least one allele shorter than 22 KIV repeats as assessed by the previously conducted size determination already available at time of sample selection, the gel concentration was 1% (2 g SeaKem LE agarose (Lonza) in 200 ml 0.5x TAE Buffer) an the following programme was used: run duration: 18h23 min; temperature: 14°C; Angle: 120°; Initial switch time: 1.73 s; Final switch time: 14.92 s Gradient: 6.00; Ramping constant: 0.

Size determination for all other samples was conducted with a gel concentration of 1.25% (2.5 g SeaKem LE agarose (Lonza) in 200 ml 0.5x TAE Buffer) by the following programme: run duration: 25 h; temperature: 14°C; Angle: 120°; Initial switch time: 7.86 s; Final switch time: 28.46 s Gradient: 6.00; Ramping constant: -1.360. 2.5g. Samples heterozygous for one allele ≤ 22 KIV repeats and one allele ≥ 35 KIV were run for size determination with both protocols.

For cutting separated alleles, two half plugs were digested according to the protocol given above. Each sample was applied twice on the same gel, which was split in two after PFGE (figure S1). The lambda ladder, applied at both sides of the gel and between the parts for Southern Blotting and cutting, was used to control for the homogeneity of running distances across the gel after staining the gel with ethidium bromide (the part for cutting the alleles was not stained, but cut beforehand). Then, on the one half the position of the bands was identified by Southern Blotting, using a KIV-2-specific probe [19], while the other half of the gel, stored in a sealed plastic bag at 4°C in the meantime, was afterwards used to cut the corresponding positions which contained the KIV-2 alleles [10]. The haploid DNA of the separated KIV-2 CNV alleles was then extracted from the gel slices by centrifugation in Ultrafree-DA Centrifugal Unit tubes (Merck Millipore).

In all samples, the difference in KIV-2 CNV size between the short and the long allele was at least 3 KIV-2 repeats (range 3 to 20, median 7 repeats size difference), which allows successful separation of alleles for sequence analysis [10].

***Protocol for cloning***

Amplicons from the PCRs “421” and “422” (table S2, figure 3) were run on 1% TAE gel in 1x TAE buffer for one hour at 120V and excised from the gel. The peqGOLD gel Extraction Kit protocol A (Peqlab; Art. Nr: 12-2501) was used for clean up of the amplicons. These were then digested with the restriction enzymes EcoRV and SalI (Promega) according to the manufacturer's recommendations and purified using peqGOLD gel Extraction Kit protocol B (Peqlab; Art. Nr: 12-2501) before ligation. T4 ligase (Promega) was used for ligation overnight at 37°C into a pENTR 3C vector (Invitrogen), which was digested with the same restriction enzymes, followed by transformation in competent bacterial cells (One Shot MAX Efficiency DH5α-T1 Chemically Competent Cells; Invitrogen) through a heat shock for 30 s at 43°C. The bacterial colonies were then allowed to grow overnight on nutrient agar plates containing Kanamycin (end concentration: 50µg/ml) at 37°C. Only colonies of bacterial cells containing the recombinant plasmid are expected to grow on these plates. Plasmid minipreps were then made using PeqGOLD plasmid miniprep Kit (Peqlab), after picking discrete colonies from the plates and growing each colony in 5ml of LB medium containing kanamycin (end concentration: 50µg/ml) at 37°C in a shaking incubator. A small volume of the purified clones was digested with the restriction enzymes (Sal I, EcoRV) and run on a 1% agarose gel 1x TAE to identify the cloned fragment before proceeding for sequence analysis.

***Protocol for Cycle Sequencing***

Purified PCR products and clones (minipreps) were used to perform cycle sequencing using ABI PRISM® BigDye® Terminator v1.1 Cycle Sequencing Kit (Applied Biosystems) for a total reaction volume of 10-14ul (depending on the amount of template used) containing 1.6ul of 10X sequencing buffer, 1ul of big dye, 6ul of Aqua bidestillata. The amount of template used was 1 ul for clones and, 1 to 5ul for the cleaned up PCR product, depending on their concentration. The thermo cycler program for the cycle sequencing reaction comprised an initial step at 96°C for 2min, followed by 50 cycles of 30sec at 96°C, 20 sec at 55° or 50°C, and 1min at 60°C. Products were cleaned up on Sephadex loaded 96well filter plates (AcroPrep) according to the manufacturer’s protocol. The cleaned up products were then sequenced on a 3130 xl Genetic Analyzer (Applied Biosystems) at the Sequencing and Genotyping Core Facility of Innsbruck Medical University. The sequences were aligned and analyzed with the SeqScape® 2.7.0 sequence alignment tool from Applied Biosystems.
