# Supplementary material for: Sequence Variation within the KIV-2 Copy Number Polymorphism of the Human LPA Gene in African, Asian, and European Populations
Source: PLoS One. 2015 Mar 30;10(3):e0121582. doi: 10.1371/journal.pone.0121582 (PMC4378929; doi:10.1371/journal.pone.0121582)
Supplement: S1 Table — PCR and sequencing primer used are given with all their annealing sites according to Homo sapiens chromosome 6, GRCh37.p13 Primary Assembly, which comprises six KIV-2 copies. For PCR primers 421U and 421U_2, seven annealing sites are present, as these primers are also annealing upstream of the non-repetitive KIV-3 exon 1, which is identical to KIV-2 exon 1 type B. However, as the lower primer for the PCR was specific for the KIV-2 region itself, KIV-3 exon 1 was not amplified by the PCR. For primer 422L, there is an additional annealing site outside the KIV-2 CNV downstream of the non-repetitive KIV-1 exon 2, which is identical to KIV-2 exon 2. Here specify of the PCR product is ensured by the KIV-2 specific primer 422U. For the primer combinations, see Fig. 3 and S2 Table. For the sequencing primers, only the annealing sites within the PCR products are shown. No SNPs are reported for any of the primer positions in the reference assembly. (DOC) [file pone.0121582.s006.doc]

**S1 Table. Primers for PCR and sequencing.**

|  | **Primer** | **Sequence 5'-3'** | **Chromosomal positions** |
| --- | --- | --- | --- |
| **PCR primers** |  |  |  |
|  | 422U | AGAAACAAACCTACTAAACCTGACAG | 161,062,500 - 161,062,475 |
|  |  |  | 161,056,957 - 161,056,932 |
|  |  |  | 161,051,408 - 161,051,383 |
|  |  |  | 161,045,864 - 161,045,839 |
|  |  |  | 161,040,318 - 161,040,293 |
|  |  |  | 161,034,766 - 161,034,741 |
|  | 422L | CACCAGAAATCACTCCGCTG | 161,061,520 - 161,061,539 |
|  |  |  | 161,055,976 - 161,055,995 |
|  |  |  | 161,050,427 - 161,050,446 |
|  |  |  | 161,044,883 - 161,044,902 |
|  |  |  | 161,039,337 - 161,039,356 |
|  |  |  | 161,033,785 - 161,033,804 |
|  |  |  | 161,067,067 - 161,067,086 |
|  | 421U | TTGGCTTTCATGATCAACG | 161,066,472 - 161,066,454 |
|  |  |  | 161,060,925 - 161,060,907 |
|  |  |  | 161,055,381 - 161,055,363 |
|  |  |  | 161,049,832 - 161,049,814 |
|  |  |  | 161,044,288 - 161,044,270 |
|  |  |  | 161,038,742 - 161,038,724 |
|  |  |  | 161,033,190 - 161,033,172 |
|  | 421L | TTTTTCTGACAATCGGAATATAC | 161,065,405 - 161,065,426 |
|  |  |  | 161,059,858 - 161,059,879 |
|  |  |  | 161,054,311 - 161,054,332 |
|  |  |  | 161,048,765 - 161,048,786 |
|  |  |  | 161,043,221 - 161,043,242 |
|  |  |  | 161,037,675 - 161,037,696 |
|  | 421U_2 | TCAGGATGCAGGGCATGAG | 161,066,618 - 161,066,600 |
|  |  |  | 161,061,071 - 161,061,053 |
|  |  |  | 161,055,527 - 161,055,509 |
|  |  |  | 161,049,978 - 161,049,958 |
|  |  |  | 161,044,434 - 161,044,416 |
|  |  |  | 161,038,888 - 161,038,870 |
|  |  |  | 161,033,336 - 161,033,318 |
| **Sequencing primers** |  |  |  |
|  | seq 422L | GCAACACTCGAGCATCCG | 161,061,589 - 161,061,606 |
|  |  |  | 161,056,045 - 161,056,062 |
|  |  |  | 161,050,496 - 161,050,513 |
|  |  |  | 161,044,952 - 161,044,969 |
|  |  |  | 161,039,406 - 161,039,423 |
|  |  |  | 161,033,854 - 161,033,871 |
|  | seq 421L | CCCAGAGAGAAAAGGCAAACG | 161,065,489 - 161,065,509 |
|  |  |  | 161,059,942 - 161,059,962 |
|  |  |  | 161,054,395 - 161,054,415 |
|  |  |  | 161,048,849 - 161,048,869 |
|  |  |  | 161,043,305 - 161,043,325 |
|  |  |  | 161,037,759 - 161,037,779 |
|  | seq 422U | GAGAGAGTGTGGGGTGC | 161,061,948 - 161,061,932 |
|  |  |  | 161,056,404 - 161,056,388 |
|  |  |  | 161,050,855 - 161,050,839 |
|  |  |  | 161,045,311 - 161,045,289 |
|  |  |  | 161,039,765 - 161,039,749 |
|  |  |  | 161,034,213 - 161,034,197 |
|  | seq 421U | TCGTAATTCTCATAGACTCC | 161,066,140 - 161,066,121 |
|  |  |  | 161,060,593 - 161,060,574 |
|  |  |  | 161,055,044 - 161,055,026 |
|  |  |  | 161,049,500 - 161,049,481 |
|  |  |  | 161,043,956 - 161,043,937 |
|  |  |  | 161,038,410 - 161,038,391 |

PCR and sequencing primer used are given with all their annealing sites according to Homo sapiens chromosome 6, GRCh37.p13 Primary Assembly, which comprises six KIV-2 copies.

For PCR primers 421U and 421U_2, seven annealing sites are present, as these primers are also annealing upstream of the non-repetitive KIV-3 exon 1, which is identical to KIV-2 exon 1 type B. However, as the lower primer for the PCR was specific for the KIV-2 region itself, KIV-3 exon 1 was not amplified by the PCR. For primer 422L, there is an additional annealing site outside the KIV-2 CNV downstream of the non-repetitive KIV-1 exon 2, which is identical to KIV-2 exon 2. Here specify of the PCR product is ensured by the KIV-2 specific primer 422U. For the primer combinations, see Fig. 3 and S2 Table.

For the sequencing primers, only the annealing sites within the PCR products are shown. No SNPs are reported for any of the primer positions in the reference assembly.
