# Supplementary material for: Sequence Variation within the KIV-2 Copy Number Polymorphism of the Human LPA Gene in African, Asian, and European Populations
Source: PLoS One. 2015 Mar 30;10(3):e0121582. doi: 10.1371/journal.pone.0121582 (PMC4378929; doi:10.1371/journal.pone.0121582)
Supplement: S2 Table — PCRs for the batchwise screening on DNA from separated alleles demanded 45 cycles, as template concentration after PFGE and cleanup of the cut gel slice was lower than for the genomic sample used for cloning. The batchwise screening of the genomic DNA from the same individual was run at 35 cycles. HOT FIREPol was purchased from Solis BioDyne; Pfu polymerase from Fermentas; and the LR PCR kit from Qiagen. (DOC) [file pone.0121582.s007.doc]

**S2 Table. PCRs conducted in our study.**

|  | **PCR** | | | | | |
| --- | --- | --- | --- | --- | --- | --- |
|  | **421** | | **422** | | **421to422** | **422to421** |
|  | **batchwise** | **cloning** | **batchwise** |  |  |  |
| **Product length** | **1069bp** | **1069bp** | **982bp** | **5105bp** | **5105bp** | **2645bp** |
| **Total volume** | 25ul | 100 ul | 25 ul | 25 ul | 25 ul | 25 ul |
| **Upper Primer** | 421U | 421U modified | 422U | 421U_2 | 421U_2 | 422U |
| **Lower Primer** | 421L | 421L modified | 422L | 422L | 422L | 421L |
| **Primer (100 µM)** | 0.25ul | 0.6 ul | 0.25 ul | 0.15 ul | 0.15 ul | 0.15 ul |
| **Polymerase** | HOT FIREPol (5U/ul) | Pfu (2.5U/µl) | HOT FIREPol (5U/ul) | LR PCR enzyme mix (5U/µl) | LR PCR enzyme mix (5U/µl) | LR PCR enzyme mix (5U/µl) |
| **Taq amount** | 0.5ul | 1.2 ul | 0.5 ul | 0.2 ul | 0.2ul | 0.2 ul |
| **Buffer type** | 10X HOT FIREPol® B1 | 10X Pfu Buffer with MgCl_2_ | 10X HOT FIREPol® B1 | 10X LR PCR buffer | 10X LR PCR buffer | 10X LR PCR buffer |
| **Buffer amount** | 2.5ul | 10ul | 2.5 ul | 2.5 ul | 2.5 ul | 2.5 ul |
| **Additional MgCl_2_** | 2.5ul | - | 2.5 ul | - | - | - |
| **dNTP Mix** | (1,25 mM each dNTP) | (1,25 mM each dNTP) | (1,25 mM each dNTP) | (10 mM each dNTP) | (10 mM each dNTP) | (10 mM each dNTP) |
| **dNTP mix amount** | 4ul | 16 ul | 4 ul | 1.25 ul | 1.25 ul | 1.25 ul |
| **Template amount** | 4-6ul | 8 ul | 4-6 ul | 4-6 ul | 4-6 ul | 4-6 ul |
| **PCR programme** |  |  |  |  |  |  |
| **Initial temperature** | 15 min at 95°C | 15 min at 95°C | 15 min at 95°C | 3 min at 93°C | 3 min at 93°C | 3 min at 93°C |
| **Number of cycles** | 45 | 35 | 45 | 45 | 45 | 45 |
| **Denaturation** | 1 min at 95°C | 1 min at 95°C | 1 min at 95°C | 15 s at 93°C | 15 s at 93°C | 15 s at 93°C |
| **Annealing** | 1 min at 62°C | 1 min at 65°C | 1 min at 66°C | 30 s at 60°C | 30 s at 60°C | 30 s at 60°C |
| **Extension** | 1 min at 72°C | 3 min at 72°C | 1 min at 72°C | 5 min at 68°C | 5 min at 68°C | 3 min at 68°C |
| **Final extension** | 10 min at 72°C | 10 min at 72°C | 10 min at 72°C | 10 min at 68°C | 10 min at 68°C | 10 min at 68°C |

*According to the reference sequence, product length varies depending on the intronic sequence between the six (I to VI) KIV-2 copies. For 421, product length varies between 1069bp (I, II, IV, V, and VI) and 1072bp (III). For 422, all fragments are 982bp, except for the first copy (I) with 981bp. For 422to421, product lengths are expected to range from 2644bp to 2648bp, with 2645bp for most copies, and for 421to442 from 5096bp to 5104bp. For chromosomal positions of primer annealing sites, see S1 Table.

PCRs for the batchwise screening on DNA from separated alleles demanded 45 cycles, as template concentration after PFGE and cleanup of the cut gel slice was lower than for the genomic sample used for cloning. The batchwise screening of the genomic DNA from the same individual was run at 35 cycles. HOT FIREPol® was purchased from Solis BioDyne; Pfu polymerase from Fermentas; and the LR PCR kit from Qiagen.
