# Supplementary material for: Sequence Variation within the KIV-2 Copy Number Polymorphism of the Human LPA Gene in African, Asian, and European Populations
Source: PLoS One. 2015 Mar 30;10(3):e0121582. doi: 10.1371/journal.pone.0121582 (PMC4378929; doi:10.1371/journal.pone.0121582)
Supplement: S3 Table — The specificity of the PCRs for the regions directly flanking the KIV-2 CNV depends on primer 412U in case of 412 (annealing site 6:161,068,509–161,068,527), and on primer 431L (annealing site 6:161,065,900–161,065,923) in case of 431. 421L also anneals in the KIV-2 exon 2 copies, and 431U in KIV-2 domains of type B. (DOC) [file pone.0121582.s008.doc]

**S3 Table. PCRs for flanking regions of the KIV-2 CNV.**

|  | **412** | **431** |
| --- | --- | --- |
| **Product length** | 1219bp | 1296bp |
| **Total volume** | 25µl | 25µl |
| **Upper Primer** | 412U (5 ‘GGAATGGAGGGTCTGGGAC 3‘) | 431U (5‘ GAGCGCACTTTGCAGTGAGAAG 3‘) |
| **Lower Primer** | 412L (5‘ CTTCTGCGTCTGAGCATTGC 3‘) | 431L (5‘ AAACTCCAATCCCTCTCCTCTGC 3‘) |
| **Primer amount (100µM)** | 0.15µl | 0.15µl |
| **Polymerase** | Qiagen HotStar (5U/µl) | Qiagen HotStar (5U/µl) |
| **Taq amount** | 0.15µl | 0.15µl |
| **Buffer type** | 10X Qiagen with MgCl2 | 10X Qiagen with MgCl2 |
| **Buffer amount** | 2.5µl | 2.5µl |
| **Additional MgCl_2_** | - | - |
| **dNTP Mix** | (1.25 mM each dNTP) | (1.25 mM each dNTP) |
| **dNTP mix amount** | 4µl | 4µl |
| **Template amount** | 4µl | 4µl |
| **Aqua bidestilata** | 14.05µl | 14.05µl |
| **PCR programme** |  |  |
| **Initial temperature** | 15 min at 95°C | 15 min at 95°C |
| **Number of cycles** | 40 | 40 |
| **Denaturation** | 1 min at 95°C | 40 sec at 95°C |
| **Annealing** | 1 min at 65°C | 40 sec at 64°C |
| **Extension** | 1 min at 72°C | 1.5 min at 72°C |
| **Final extension** | 10 min at 72°C | 10 min at 72°C |

The specificity of the PCRs for the regions directly flanking the KIV-2 CNV depends on primer 412U in case of 412 (annealing site 6:161,068,509- 161,068,527), and on primer 431L (annealing site 6: 161,065,900 - 161,065,923) in case of 431. 421L also anneals in the KIV-2 exon 2 copies, and 431U in KIV-2 domains of type B.
