# Supplementary material for: Sequence Variation within the KIV-2 Copy Number Polymorphism of the Human LPA Gene in African, Asian, and European Populations
Source: PLoS One. 2015 Mar 30;10(3):e0121582. doi: 10.1371/journal.pone.0121582 (PMC4378929; doi:10.1371/journal.pone.0121582)
Supplement: S4 Table — Sequence variation in KIV-2 exon 1 (K421) retrieved from Ensembl (ENSG00000198670; GRCh37) and previous studies in comparison to our findings from the cloning and batchwise screening. The data from external databases are given for populations of African and European descent, for comparison to our study. In the external databases, no variants were reported for Asians. The chromosomal positions shown for variants found in our own study or reported by Rosby et al. (2000) and Parson et al. (2004) are shown only according to the first KIV-2 copy in the reference sequence, as they could be present on any of the KIV-2 copies in the LPA alleles screened. Further possible coding positions can be allocated by adding multitudes of 342 bases (as the length of one KIV-2 repeat is 160 bp for exon 1 and 182 bp for exon 2), depending on the length of the KIV-2 CNV in any individual LPA allele. KIV-2 exon 1 is labeled as ‘K421’. The Roman numerals (I to VI) with K421 indicate the order of KIV-2 copies in the reference sequence. Chromosomal positions are given as they are entered in the database. Bases are given as on the reverse strand. As the third KIV-2 (K421 III) is the only copy of type B in the reference sequence, sequence reads from KIV-2 type A copies could be entered as variants at the exonic positions 14, 41, and 86 when aligned to the type B copy, and vice versa. Possibly for this latter reason, the frequencies reported by the ClinSeq panel for K421 I, II, IV, V, and VI appear questionable. Additional complication and potential misallocation could also stem from the complete sequence homology between KIV-3 exon 1 and KIV-2 exon 1 type B. A variable site at position 86 of the non-repetitive KIV-3 exon 1 is reported as SNP c.2529T>C (as rs113727842, rs80331546, or rs3798222). Its minor allele frequencies are similar to those reported by the ClinSeq panel for their entries listed above. Hence, we did not include this variation in our discussion. (DOC) [file pone.0121582.s009.doc]

**S4 Table. Comparison of own results with external data on variants in KIV-2 exon 1.**

| **Source** | **Region** | **Position** | | | **rs number** | **Variant** | **MAF (Allele count)** | **Population** | **Panel** | **Mutation Type** |
| --- | --- | --- | --- | --- | --- | --- | --- | --- | --- | --- |
|  |  | **Exonic** | **Chromosomal*** | **Coding*** |  |  |  |  |  |  |
| ***Ensembl*** | K421 I | 4 | 161066035 | c.395 | rs146038804 | C>T | Not given |  |  | p.Pro132Leu |
|  | K421 I | 86 | 161065953 | c.477 | rs199542240 | A>C | 0.608 (A: 112, C: 174) | European | CLINSEQ-SNP | synonymous |
|  |  |  |  |  | rs3798222 | T>C |  |  |  |  |
|  | K421 I | 103 | 161065936 | c.494 | rs201240804 | C>T | 0.001 (C: 1053, T: 1) | European | CLINSEQ-SNP | p.Ala165Val |
|  |  |  |  |  | rs9347424 | C>A |  |  |  |  |
|  | K421 II | 86 | 161060406 | c.819 | rs202053533 | A>C | 0.630 (A: 96; C: 163) | European | CLINSEQ-SNP | synonymous |
|  |  |  |  |  | rs3798222 | T>C |  |  |  |  |
|  | K421 II | 103 | 161060389 | c.836 | rs201125696 | C>T | 0.001 (C:1081, A: 1) | European | CLINSEQ-SNP | p.Ala279Val |
|  |  |  |  |  | rs9347424 | C>A |  |  |  |  |
|  | K421 III | 41 | 161054904 | c.1116 | [rs373100474](http://www.ncbi.nlm.nih.gov/projects/SNP/snp_ref.cgi?searchType=adhoc_search&type=rs&rs=rs373100474) | C>T | 0.001 (C: 2066, T: 2) | African-American | ESP6500 | synonymous |
|  |  |  |  |  |  |  | 0.0005 (C: 4543, T: 1) | European-American | ESP6500 |  |
|  | K421 III | 53 | 161054892 | c.1128 | rs150948997 | C>T | Not given |  |  | synonymous |
|  | K421 III | 71 | 161054874 | c.1146 | rs202217385 | C>T | 0.001 (C: 1296, T: 1) | European | CLINSEQ-SNP | synonymous |
|  | K421 III | 86 | 161054859 | c.1161 | rs4709450 | T>C/A | Not given | Not given |  | synonymous |
|  |  |  |  |  | rs3798222 | T>C |  |  |  |  |
|  |  |  |  |  | rs80331546 | T>C |  |  |  |  |
|  | K421 III | 133 | 161054812 | c.1208 | rs201102026 | G>T | 0.001 (G: 1316, T: 1) | European | CLINSEQ-SNP | p.Ser403Ile |
|  | K421 IV | 86 | 161049313 | c.1503 | rs201591386 | A>C | 0.601 (A: 126, C: 189) | European | CLINSEQ-SNP | synonymous |
|  |  |  |  |  | rs3798222 | T>C |  |  |  |  |
|  | K421 IV | 103 | 161049296 | c.1520 | rs201139907 | C>T | 0.001 (C: 1092, T: 1) | European | CLINSEQ-SNP | p.Ala507Val |
|  |  |  |  |  | rs9347424 | C>A |  |  |  |  |
|  | K421 V | 86 | 161043769 | c.1845 | rs201719709 | A>C | 0.596 (A: 110, C: 161) | European | CLINSEQ-SNP | synonymous |
|  |  |  |  |  | rs3798222 | T>C |  |  |  |  |
|  | K421 V | 92 | 161043763 | c.1851 | rs200723881 | A>G | 0.002 (A: 615, C: 1) | European | CLINSEQ-SNP | synonymous |
|  | K421 V | 103 | 161043752 | c.1862 | rs199760470 | C>T | 0.001 (C: 1061, A: 1) | European | CLINSEQ-SNP | p.Ala621Val |
|  |  |  |  |  | rs9347424 | C>A |  |  |  |  |
|  | K421 VI | 86 | 161038223 | c.2187 | rs199622959 | A>C | 0.636 (A: 104, C: 182) | European | CLINSEQ-SNP | synonymous |
|  |  |  |  |  | rs3798222 | T>C |  |  |  |  |
|  | K421 VI | 103 | 161038206 | c.2204 | rs201064428 | C>T | 0.001 (C: 1077, T: 1) | European | CLINSEQ-SNP | p.Ala735Val |
|  |  |  |  |  | rs9347424 | C>A |  |  |  |  |
| **Parson et al.^1^** | K421 | 7 | 161066032 | c.398 |  | C>T | 1/93 clones | European |  | p.Thr133Ile |
|  | K421 | 10 | 161066029 | c.401 |  | A>G | 1/93 clones | European |  | p.Glu134Gly |
|  | K421 | 14 | 161066025 | c.405 |  | A>G | 32/93 clones | European |  | synonymous |
|  | K421 | 33 | 161066006 | c.424 |  | T>C | 1/93 clones | European |  | p.Cys142Arg |
|  | K421 | 34 | 161066005 | c.425 |  | G>A | 1/93 clones | European |  | p.Cys142Tyr |
|  | K421 | 35 | 161066004 | c.426 |  | C>A | 1/93 clones | European |  | p.Cys142Ter |
|  | K421 | 41 | 161065998 | c.432 |  | T>C | 32/93 clones | European |  | synonymous |
|  | K421 | 52 | 161065987 | c.443 |  | A>G | 1/93 clones | European |  | p.Gln148Arg |
|  | K421 | 60 | 161065979 | c.451 |  | C>T | 9/93 clones | European |  | p.Arg151Ter |
|  | K421 | 62 | 161065977 | c.453 |  | A>G | 1/93 clones | European |  | synonymous |
|  | K421 | 69 | 161065970 | c.460 |  | T>C | 1/93 clones | European |  | p.Tyr154His |
|  | K421 | 86 | 161065953 | c.477 |  | A>T/G | 27/93 clones | European |  | synonymous |
|  | K421 | 101 | 161065938 | c.492 |  | A>G | 1/93 clones | European |  | synonymous |
|  | K421 | 146 | 161065893 | c.537 |  | A>G | 1/93 clones | European |  | synonymous |
| **Rosby et al.^2^** | K421 | 20 | 161066019 | c.411 |  | T>C | 1/ 39 clones | Norwegian |  | synonymous |
|  | K421 | 25 | 161066014 | c.416 |  | T>C | 1/39 clones | Norwegian |  | p.Val139Ala |
|  | K421 | 75 | 161065964 | c.466 |  | A>T | 1/39 clones | Norwegian |  | p.Thr156Ser |
|  | K421 | 112 | 161065927 | c.503 |  | C>T | 1/39 clones | Norwegian |  | p.Ser168Phe |
| **McLean et al.^3^** | K421 | 14 | 161066025 | c.405 |  | A>G | 4/28 KIV-2 copies | cDNA (1 individual) |  | synonymous |
|  | K421 | 41 | 161065998 | c.432 |  | T>C | 4/28 KIV-2 copies | cDNA (1 individual) |  | synonymous |
|  | K421 | 86 | 161065953 | c.477 |  | A>T | 4/28 KIV-2 copies | cDNA (1 individual) |  | synonymous |
| **Own data** |  |  |  |  |  |  |  |  |  |  |
| batchwise | K421 | 14 | 161066025 | c.405 |  | A>G | 3/50 alleles | African |  | synonymous |
|  |  |  |  |  |  |  | 3/20 alleles | Austrian |  |  |
|  |  |  |  |  |  |  | 14/20 alleles | Chinese |  |  |
|  | K421 | 20 | 161066019 | c.411 |  | T>C | 1/ 90 alleles | Khoi San |  | synonymous |
|  | K421 | 41 | 161065998 | c.432 |  | T>C | 3/50 alleles | African |  | synonymous |
|  |  |  |  |  |  |  | 3/20 alleles | Austrian |  |  |
|  |  |  |  |  |  |  | 14/20 alleles | Chinese |  |  |
|  | K421 | 69 | 161065970 | c.460 |  | T>C | 1/ 90 alleles | Khoi San |  | p.Tyr154His |
|  | K421 | 86 | 161065953 | c.477 |  | A>T | 1/50 alleles | African |  | synonymous |
|  |  |  |  |  |  |  | 2/20 alleles | Austrian |  |  |
|  |  |  |  |  |  |  | 10/20 alleles | Chinese |  |  |
|  | K421 | 113 | 161065926 | c.504 |  | T>C | 1/ 90 alleles | South African |  | synonymous |

*: Only assigned to genomic positions in specific KIV-2 copies in Ensembl; otherwise given as for the first KIV-2 copy in the reference sequence

^1^: [10]; ^2^: [13]; ^3^: [2]

Sequence variation in KIV-2 exon 1 (K421) retrieved from Ensembl (ENSG00000198670; GRCh37) and previous studies in comparison to our findings from the cloning and batchwise screening. The data from external databases are given for populations of African and European descent, for comparison to our study. In the external databases, no variants were reported for Asians. The chromosomal positions shown for variants found in our own study or reported by Rosby *et al.* (2000) and Parson *et al.* (2004) are shown only according to the first KIV-2 copy in the reference sequence, as they could be present on any of the KIV-2 copies in the *LPA* alleles screened. Further possible coding positions can be allocated by adding multitudes of 342 bases (as the length of one KIV-2 repeat is 160bp for exon 1 and 182 bp for exon 2), depending on the length of the KIV-2 CNV in any individual *LPA* allele.

KIV-2 exon 1 is labeled as ‘K421’. The Roman numerals (I to VI) with K421 indicate the order of KIV-2 copies in the reference sequence. Chromosomal positions are given as they are entered in the database. Bases are given as on the reverse strand. As the third KIV-2 (K421 III) is the only copy of type B in the reference sequence, sequence reads from KIV-2 type A copies could be entered as variants at the exonic positions 14, 41, and 86 when aligned to the type B copy, and vice versa.

Possibly for this latter reason, the frequencies reported by the ClinSeq panel for K421 I, II, IV, V, and VI appear questionable. Additional complication and potential misallocation could also stem from the complete sequence homology between KIV-3 exon 1 and KIV-2 exon 1 type B. A variable site at position 86 of the non-repetitive KIV-3 exon 1 is reported as SNP c.2529T>C (as rs113727842, [rs80331546](http://browser.1000genomes.org/Homo_sapiens/Variation/Population?db=core;g=ENSG00000198670;r=6:160952515-161087407;v=rs80331546;vdb=variation;vf=24115180), or [rs3798222](http://browser.1000genomes.org/Homo_sapiens/Variation/Population?db=core;g=ENSG00000198670;r=6:160952515-161087407;v=rs3798222;vdb=variation;vf=2858683)). Its minor allele frequencies are similar to those reported by the ClinSeq panel for their entries listed above. Hence, we did not include this variation in our discussion.
