# Supplementary material for: Sequence Variation within the KIV-2 Copy Number Polymorphism of the Human LPA Gene in African, Asian, and European Populations
Source: PLoS One. 2015 Mar 30;10(3):e0121582. doi: 10.1371/journal.pone.0121582 (PMC4378929; doi:10.1371/journal.pone.0121582)
Supplement: S5 Table — Sequence variation in KIV-2 exon 2 (K422) retrieved from Ensembl (ENSG00000198670; GRCh37) and previous studies in comparison to our findings from the cloning and batchwise screening. The data from external databases are given for populations of African and European descent, for comparison to our study. In the external databases, no variants were reported for Asians. KIV-2 exon 2 is labeled as ‘K422’. The Roman numerals indicate the order of KIV-2 copies in the reference sequence. For the data retrieved via Ensembl, the chromosomal positions are given as they are entered in the database. In the current build of the reference sequence, any variation reported in Ensemble for K422 is either assigned to the second or fifth KIV-2 copy. The chromosomal positions shown for variants found in our own study or reported by Rosby et al. (2000) and Parson et al. (2004) are shown only according to the first KIV-2 copy in the reference sequence, as they could be present on any of the KIV-2 copies in the LPA alleles screened. Further possible coding positions can be allocated by adding multitudes of 342 bases (as the length of one KIV-2 repeat is 160 bp for exon 1 and 182 bp for exon 2), depending on the length of the KIV-2 CNV in any individual LPA allele. (DOC) [file pone.0121582.s010.doc]

**S5 Table. Comparison of own results with external data on variants in KIV-2 exon 2**

| **Source** | **Region** | **Position** | | | **rs number** | **Variant** | **MAFs (Allele count)** | **Population** | **Panel** | **Mutation Type** |
| --- | --- | --- | --- | --- | --- | --- | --- | --- | --- | --- |
|  |  | **Exonic** | **Chromosomal*** | **Coding*** |  |  |  |  |  |  |
|  | K422 I | 27 | 161061854 |  | ss1323156587 | A>G | Not given |  |  | p.Asn193Ser |
|  | K422 I | 155 | 161061726 |  | ss1323156586 | C>T | 0.01 (C: 1309, T 13) | Africans | 1000 Genomes | p.Pro236Ser |
|  | K422 II | 58 | 161056279 | c.951 | rs200223332 | T>C | *0.023 (T: 4894, C: 114)* | *All populations* | 1000 Genomes | synonymous |
|  |  |  |  |  |  |  | 0.003 (T: 692, C: 2) | Admixed Americans | 1000 Genomes |  |
|  |  |  |  |  |  |  | 0.085 (T: 1210, C: 112) | Africans | 1000 Genomes |  |
|  | K422 II | 101 | 161056236 | c.994 | rs201244072 | C>G | *0.002 (C: 4996, G: 12)* | *All populations* | 1000 Genomes | p.Gln332Glu |
|  |  |  |  |  |  |  | 0.009 (C: 1310, G: 12) | Africans | 1000 Genomes |  |
|  |  |  |  |  |  |  | 0.003 (C: 2582, G: 8) | African-Americans | ESP 6500 |  |
|  | K422 II | 113 | 161056224 | c.1006 | rs201736559 | G>A | *0.002 (G: 5000, A: 8)* | *All populations* | 1000 Genomes | p.Ala336Thr |
|  |  |  |  |  |  |  | 0.006 (G: 1314, A: 8) | Africans | 1000 Genomes |  |
|  |  |  |  |  |  |  | 0.005 (G: 2636, A: 12) | African-Americans | ESP 6500 |  |
|  | K422 II | 150 | 161056187 | c.1043 | rs200465199 | C>T | 0.002 (C: 2956, T: 6) | African-Americans | ESP 6500 | p.Pro348Leu |
|  |  |  |  |  |  |  | 0.007 (C: 7211, T: 51) | European-Americans | ESP 6500 |  |
|  | K422 II | 160 | 161056177 |  | ss1323156575 | C>A | 0.001 (C:1321, A: 1) | Africans |  | p.Ser 351Arg |
|  | K422 II | 170 | 161056167 | c.1063 | rs62442779 | C>T | in 1 individual | Not given | Genome resequencing | p.Pro355Ser |
|  | K422 II | 171 | 161056166 | c.1064 | rs62442778 | C>T | in 1 individual | Not given | Genome resequencing | p.Pro355Ser |
|  | K422 V | 150 | 161039548 | c.2069 | rs200607038 | C>T | 0.004 (C: 277, T: 1) | Europeans | CLINSEQ-SNP | p.Pro690Leu |
| **Parson et al.^1^** | K422 |  |  |  |  | none |  | European | 93 clones |  |
| **Rosby et al.^2^** | K422 | 8 | 161061873 | c.559 |  | A>G | 1/34 clones | Norwegian |  | p.Ile187Val |
|  | K422 | 18 | 161061863 | c.569 |  | A>G | 1/34 clones | Norwegian |  | p.Tyr190Cys |
|  | K422 | 34 | 161061847 | c.585 |  | T>C | 1/34 clones | Norwegian |  | synonymous |
|  | K422 | 117 | 161061764 | c.668 |  | A>T | 1/34 clones | Norwegian |  | p.Glu223Val |
|  | K422 | 118 | 161061763 | c.669 |  | A>T | 1/34 clones | Norwegian |  | synonymous |
|  | K422 | 158 | 161061723 | c.709 |  | A>G | 2/34 clones | Norwegian |  | p.Ser237Gly |
| **McLean et al.^3^** | K422 |  |  |  |  | none |  | cDNA (1 individual) | 28 KIV-2 copies |  |
| **Own data** | K422 |  |  |  |  |  |  |  |  |  |
| Cloning | K422 | 31 | 161061850 | c.582 |  | A>T | 9/159 clones | South African |  | synonymous |
|  | K422 | 58 | 161061823 | c.609 |  | T>C | 9/159 clones | South African |  | synonymous |
| Batchwise | K422 | 113 | 161061768 | c.664 |  | G>A | 2/90 alleles | Khoi San, Gabonese |  | p.Ala222Thr |

*: Only assigned to genomic positions in specific KIV-2 copies in Ensembl; otherwise given as for the first KIV-2 copy in the reference sequence

^1^: [10]; ^2^: [13]; ^3^: [2]

Sequence variation in KIV-2 exon 2 (K422) retrieved from Ensembl (ENSG00000198670; GRCh37) and previous studies in comparison to our findings from the cloning and batchwise screening. The data from external databases are given for populations of African and European descent, for comparison to our study. In the external databases, no variants were reported for Asians.

KIV-2 exon 2 is labeled as ‘K422’. The Roman numerals indicate the order of KIV-2 copies in the reference sequence. For the data retrieved via Ensembl, the chromosomal positions are given as they are entered in the database. In the current build of the reference sequence, any variation reported in Ensemble for K422 is either assigned to the second or fifth KIV-2 copy. The chromosomal positions shown for variants found in our own study or reported by Rosby *et al.* (2000) and Parson *et al.* (2004) are shown only according to the first KIV-2 copy in the reference sequence, as they could be present on any of the KIV-2 copies in the *LPA* alleles screened. Further possible coding positions can be allocated by adding multitudes of 342 bases (as the length of one KIV-2 repeat is 160bp for exon 1 and 182 bp for exon 2), depending on the length of the KIV-2 CNV in any individual *LPA* allele.
