# Supplementary material for: Sequence Variation within the KIV-2 Copy Number Polymorphism of the Human LPA Gene in African, Asian, and European Populations
Source: PLoS One. 2015 Mar 30;10(3):e0121582. doi: 10.1371/journal.pone.0121582 (PMC4378929; doi:10.1371/journal.pone.0121582)
Supplement: S6 Table — The results from the batchwise screening for the 421 amplicon, containing the first exon of KIV-2 (K421), and the 422 amplicon, containing the second exon of KIV-2 (K422), are entered by population. Alleles are listed with their sample IDs, with Roman numerals representing the short (I) and long (II) alleles of the same sample respectively. Variable sites are named after their position relative to the start of the exon (for the chromosomal positions, see S4 and S5 Tables). For all variants the ratios of wild type to variant alleles carrying KIV-2 copies are given, as estimated by the relative height of the two peaks in the electropherogram. The KIV-CNV allele size is entered as KIV-2 plus the 9 non-repetitive KIV domains. The variable sites at positions 14, 41, and 86 of K421 differentiate KIV-2 exon 1 types A, B, and C, with the wild type allele representing type A. Allele associated Lp(a) concentration are given as derived from densitometric evaluation of Western Blots, where available, and as total Lp(a) of the individual otherwise (indicated by brackets). (DOC) [file pone.0121582.s011.doc]

**S6 Table. Variation found by batchwise sequencing of 421 and 422 amplicons**

| **Population** | **KIV CNV size of allele** | **Lp(a) [mg/dl]** | **ID of Samples carrying the haplotype** | **Bases at variable site** | | | | | | | | | |
| --- | --- | --- | --- | --- | --- | --- | --- | --- | --- | --- | --- | --- | --- |
|  |  |  |  | **Amplicon 421** | | | | | | | **Amplicon 422** | | |
|  |  |  |  | **K421 14A>G^1,2^** | **K421 20T>C** | **K421 41T>C^1,2^** | **K421 69T>C*** | **K421 86A>T^1^** | **K421 113T>C** | **K421 +1G>A** | **K422 -17C>G** | **K422 -6T>G** | **K422 113G>A^§^** |
|  |  |  | **Ref. Seq** | **A** | **T** | **T** | **T** | **A** | **T** | **G** | **C** | **T** | **G** |
| **Khoi San** | **29** | **18.9** | **K1 I** | A | T | T | T | A | T | G | C | T | G |
|  | **35** | **0** | **K1 II** | A | T | T | T | A | T | G | C | T | G |
|  | **23** | **4.4** | **K2 I** | A | T | T | T | A | T | G | C | T | G |
|  | **28** | **84.0** | **K2 II** | A | T | T | T | A | T | G | C | T | G |
|  | **19** | **12.6** | **K3 I** | A | T | T | T | A | T | G | C | 65%T;35%G | G |
|  | **26** | **18.9** | **K3 II** | A | T | T | T | A | T | G | C | T | G |
|  | **20** | **19.6** | **K4 I** | 95%A;5%G | T | 95%T;5%C | T | A | T | G | C | T | G |
|  | **26** | **19.6** | **K4 II** | A | T | T | T | A | T | G | C | T | G |
|  | **15** | **0** | **K5 I** | A | T | T | T | A | T | 85%G;15%A | C | T | 85%G;15%A |
|  | **23** | **22,7** | **K5 II** | A | 85%T;15%C | T | 90%T;10%C | A | T | G | 70%C;30%G | T | G |
| **Gabonese**  **Bantu** | **28** | **2,3** | **G1 I** | A | T | T | T | A | T | G | C | T | G |
|  | **32** | **2,3** | **G1 II** | A | T | T | T | A | T | G | C | T | G |
|  | **20** | **152.1** | **G2 I** | A | T | T | T | A | T | G | C | T | G |
|  | **27** | **3.1** | **G2 II** | A | T | T | T | A | T | G | C | T | G |
|  | **17** | **80.0** | **G3 I** | A | T | T | T | A | T | G | C | T | G |
|  | **23** | **53.4** | **G3 II** | 70%A;30%G | T | 55%T;45%C | T | 80%A;20%T | T | G | C | T | G |
|  | **29** | **10.5** | **G4 I** | A | T | T | T | A | T | G | C | T | G |
|  | **37** | **1.9** | **G4 II** | A | T | T | T | A | T | G | C | T | G |
|  | **19** | **11.9** | **G5 I** | A | T | T | T | A | T | G | C | 60%T;40%G | G |
|  | **29** | **0.6** | **G5 II** | A | T | T | T | A | T | G | C | T | G |
|  | **25** | **17.3** | **G6 I** | A | T | T | T | A | T | G | C | T | G |
|  | **28** | **21.2** | **G6 II** | A | T | T | T | A | T | G | C | T | G |
|  | **22** | **17.7** | **G7 I** | A | T | T | T | A | T | G | C | T | G |
|  | **26** | **26.5** | **G7 II** | A | T | T | T | A | T | G | C | T | 90%G;10%A |
|  | **24** | **29.5** | **G8 I** | 95%A;5%G | T | 95%T;5%C | T | A | T | G | C | T | G |
|  | **30** | **0** | **G8 II** | A | T | T | T | A | T | 95%G;5%A | C | T | G |
|  | **24** | **31.1** | **G9 I** | A | T | T | T | A | T | G | C | T | G |
|  | **31** | **13.3** | **G9 II** | A | T | T | T | A | T | G | C | T | G |
|  | **26** | **37.1** | **G10 I** | A | T | T | T | A | T | G | C | T | G |
|  | **34** | **0** | **G10 II** | A | T | T | T | A | T | G | C | 50%T;50%G | G |
| **South African Bantu** | **27** | **2.3** | **S1 I** | A | T | T | T | A | T | G | C | T | G |
|  | **41** | **0.1** | **S1 II** | A | T | T | T | A | T | G | C | T | G |
|  | **18** | **63.0** | **S2 I** | A | T | T | T | A | T | G | C | T | G |
|  | **30** | **11.1** | **S2 II** | A | T | T | T | A | T | G | C | T | G |
|  | **19** | **7.7** | **S3 I** | A | T | T | T | A | T | G | C | 50%T;50%G | G |
|  | **39** | **0** | **S3 II** | A | T | T | T | A | T | G | C | T | G |
|  | **19** | **4.6** | **S4 I** | A | T | T | T | A | T | G | C | 60%T;40%G | G |
|  | **30** | **6.9** | **S4 II** | A | T | T | T | A | 85%T;15%C | G | C | T | G |
|  | **22** | **21.3** | **S5 I** | A | T | T | T | A | T | G | C | T | G |
|  | **29** | **9.1** | **S5 II** | A | T | T | T | A | T | G | C | T | G |
| **Egyptians** | **17** | **1.8** | **E1 I** | A | T | T | T | A | T | G | C | 50%T;50%G | G |
|  | **29** | **21.8** | **E1 II** | A | T | T | T | A | T | G | C | T | G |
|  | **19** | **0.3** | **E2 I** | A | T | T | T | A | T | G | C | 60%T;40%G | G |
|  | **29** | **33.4** | **E2 II** | A | T | T | T | A | T | G | C | T | G |
|  | **14** | **7.9** | **E3 I** | A | T | T | T | A | T | G | C | T | G |
|  | **20** | **18.4** | **E3 II** | A | T | T | T | A | T | G | C | 60%T;40%G | G |
|  | **21** | **11.4** | **E4 I** | A | T | T | T | A | T | G | C | T | G |
|  | **30** | **34.1** | **E4 II** | A | T | T | T | A | T | G | C | T | G |
|  | **19** | **29.5** | **E5 I** | A | T | T | T | A | T | G | C | 70%T;30%G | G |
|  | **29** | **3.3** | **E5 II** | A | T | T | T | A | T | G | C | T | G |
| **Austrians** | **21** | **94.8** | **A1 I** | A | T | T | T | A | T | G | C | T | G |
|  | **28** | **0** | **A1 II** | A | T | T | T | A | T | G | C | T | G |
|  | **23** | **6.1** | **A2 I** | A | T | T | T | A | T | G | C | T | G |
|  | **30** | **0** | **A2 II** | A | T | T | T | A | T | G | C | T | G |
|  | **27** | **0.6** | **A3 I** | A | T | T | T | A | T | G | C | T | G |
|  | **33** | **5.7** | **A3 II** | A | T | T | T | A | T | G | C | T | G |
|  | **19** | **3.1** | **A4 I** | A | T | T | T | A | T | G | C | T | G |
|  | **32** | **3.1** | **A4 II** | A | T | T | T | A | T | G | C | T | G |
|  | **15** | **39.6** | **A5 I** | A | T | T | T | A | T | G | C | T | G |
|  | **28** | **24.6** | **A5 II** | A | T | T | T | A | T | G | C | T | G |
|  | **28** | **0.4** | **A6 I** | A | T | T | T | A | T | 95%G;5%A | C | T | G |
|  | **33** | **1.5** | **A6 II** | A | T | T | T | A | T | G | C | T | G |
|  | **28** | **0.6** | **A7 I** | 90%A;10%G | T | 85%T;15%C | T | 95%A;5%T | T | G | C | T | G |
|  | **33** | **2.6** | **A7 II** | 80%A;20%G | T | 60%T;40%C | T | 80%A;20%T | T | G | C | T | G |
|  | **29** | **31.7** | **A8 I** | A | T | T | T | A | T | G | C | T | G |
|  | **36** | **0** | **A8 II** | A | T | T | T | A | T | G | C | T | G |
|  | **22** | **7.6** | **A9 I** | A | T | T | T | A | T | G | C | T | G |
|  | **32** | **0** | **A9 II** | A | T | T | T | A | T | G | C | T | G |
|  | **16** | **59.1** | **A10 I** | A | T | T | T | A | T | G | C | T | G |
|  | **28** | **0** | **A10 II** | A | T | T | T | A | T | G | C | T | G |
| **Chinese** | **32** | **(2.4)** | **H1 I** | 75%A;25%G | T | 60%T;40%C | T | 75%A;25%T | T | G | C | T | G |
|  | **41** | **(2.4)** | **H1 II** | 75%A;25%G | T | 60%T;40%C | T | 75%A;25%T | T | G | C | T | G |
|  | **31** | **6.7** | **H2 I** | 75%A;25%G | T | 65%T;35%C | T | 75%A;25%T | T | G | C | T | G |
|  | **34** | **4.5** | **H2 II** | 75%A;25%G | T | 65%T;35%C | T | 75%A;25%T | T | G | C | T | G |
|  | **29** | **14.8** | **H3 I** | 75%A;25%G | T | 65%T;35%C | T | 70%A;30%T | T | G | C | T | G |
|  | **33** | **4.9** | **H3 II** | 75%A;25%G | T | 60%T;40%C | T | 75%A;25%T | T | G | C | T | G |
|  | **28** | **5.5** | **H4 I** | A | T | T | T | A | T | G | C | T | G |
|  | **36** | **0** | **H4 II** | A | T | T | T | A | T | G | C | T | G |
|  | **31** | **16.8** | **H5 I** | 90%A;10%G | T | 85%T;15%C | T | 95%A;5%T | T | G | C | T | G |
|  | **34** | **0** | **H5 II** | 95%A;5%G | T | 90%T;10%C | T | A | T | G | C | T | G |
|  | **20** | **88.6** | **H6 I** | A | T | T | T | A | T | G | C | T | G |
|  | **31** | **29.5** | **H6 II** | 85%A;15%G | T | 70%T;30%C | T | 80%A;20%T | T | G | C | T | G |
|  | **18** | **0** | **H7 I** | A | T | T | T | A | T | G | C | T | G |
|  | **24** | **37.9** | **H7 II** | 95%A;5%G | T | 90%T;10%C | T | A | T | G | C | T | G |
|  | **33** | **7.9** | **H8 I** | 80%A;20%G | T | 70%T;30%C | T | 90%A;10%T | T | G | C | T | G |
|  | **39** | **4.3** | **H8 II** | A | T | T | T | A | T | G | C | T | G |
|  | **27** | **25.4** | **H9 I** | 95%A;5%G | T | 95%T;5%C | T | A | T | G | C | T | G |
|  | **34** | **0** | **H9 II** | 75%A;25%G | T | 60%T;40%C | T | 70%A;30%T | T | G | C | T | G |
|  | **25** | **(94.8)** | **H10 I** | 95%A;5%G | T | 95%T;5%C | T | A | T | G | C | T | G |
|  | **35** | **(94.8)** | **H10 II** | A | T | T | T | A | T | G | C | T | G |

^1^: variant bases at these three positions together as a haplotype define KIV-2 type B; ^2^: variant bases at these two positions together as a haplotype define KIV-2 type C; *: non-synonymous variant Tyr🡪His (p.Tyr154His); ^§^: non-synonymous variant Ala 🡪Thr (p.Ala222Thr)

The results from the batchwise screening for the 421 amplicon, containing the first exon of KIV-2 (K421), and the 422 amplicon, containing the second exon of KIV-2 (K422), are entered by population. Alleles are listed with their sample IDs, with Roman numerals representing the short (I) and long (II) alleles of the same sample respectively. Variable sites are named after their position relative to the start of the exon (for the chromosomal positions, see S4 Table and S5 Table). For all variants the ratios of wild type to variant alleles carrying KIV-2 copies are given, as estimated by the relative height of the two peaks in the electropherogram. The KIV-CNV allele size is entered as KIV-2 plus the 9 non-repetitive KIV domains. The variable sites at positions 14, 41, and 86 of K421 differentiate KIV-exon 1 types A, B, and C, with the wild type allele representing type A. Allele associated Lp(a) concentration are given as derived from densitometric evaluation of Western Blots, where available, and as total Lp(a) of the individual otherwise (indicated by brackets).
